# Co-encapsulation and co-transplantation of mesenchymal stem cells reduces pericapsular fibrosis and improves encapsulated islet survival and function when allografted

**DOI:** 10.1038/s41598-017-10359-1

**Published:** 2017-08-30

**Authors:** Vijayaganapathy Vaithilingam, Margaret D. M. Evans, Denise M. Lewy, Penelope A. Bean, Sumeet Bal, Bernard E. Tuch

**Affiliations:** 1 Biomedical Manufacturing Research Program, Commonwealth Scientific and Industrial Research Organization (CSIRO), Manufacturing Flagship, North Ryde, New South Wales, Australia; 2 Australian Foundation for Diabetes Research, Sydney, New South Wales, Australia, previously at CSIRO Manufacturing Flagship, North Ryde, New South Wales, Australia

## Abstract

Pericapsular fibrotic overgrowth (PFO) is associated with poor survival of encapsulated islets. A strategy to combat PFO is the use of mesenchymal stem cells (MSC). MSC have anti-inflammatory properties and their potential can be enhanced by stimulation with proinflammatory cytokines. This study investigated whether co-encapsulation or co-transplantation of MSC with encapsulated islets would reduce PFO and improve graft survival. Stimulating MSC with a cytokine cocktail of IFN-γ and TNF-α enhanced their immunosuppressive potential by increasing nitric oxide production and secreting higher levels of immunomodulatory cytokines. *In vitro*, co-encapsulation with MSC did not affect islet viability but significantly enhanced glucose-induced insulin secretion. *In vivo*, normoglycemia was achieved in 100% mice receiving islets co-encapsulated with stimulated MSC as opposed to 71.4% receiving unstimulated MSC and only 9.1% receiving encapsulated islets alone. Microcapsules retrieved from both unstimulated and stimulated MSC groups had significantly less PFO with improved islet viability and function compared to encapsulated islets alone. Levels of peritoneal immunomodulatory cytokines IL-4, IL-6, IL-10 and G-CSF were significantly higher in MSC co-encapsulated groups. Similar results were obtained when encapsulated islets and MSC were co-transplanted. In summary, co-encapsulation or co-transplantation of MSC with encapsulated islets reduced PFO and improved the functional outcome of allotransplants.

## Introduction

Microencapsulation of pancreatic islets in alginate hydrogels is a strategy being explored as a potential cellular therapy for type 1 diabetes without the need for toxic immunosuppression. Allo- and xeno- transplantation of microencapsulated islets shows promise in the preclinical setting with blood glucose levels being normalized for extended periods in diabetic animals^1^. However, such outcomes have yet to be achieved in the clinical setting^2–4^. Graft retrieval from human recipients show the presence of dense pericapsular fibrotic overgrowth (PFO) with necrotic islets^2^ despite the administration of immunosuppression^4^. PFO is a result of host inflammatory response to antigens shed by encapsulated allogeneic/xenogeneic tissue^5, 6^. It forms a physical barrier, mainly of macrophages and fibroblasts, that prevents the transport of oxygen and other nutrients, leading to starvation, hypoxia and ultimately to islet death^7–9^. Thus, strategies aimed at reducing or preventing PFO should enhance encapsulated islet survival and improve transplantation outcomes.

Approaches to reduce PFO include altering alginate composition and chemistry^10–12^, surface modification of alginate microcapsules^13–15^, co-encapsulation with immunomodulatory Sertoli cells^16^, transplantation at different anatomical sites^17^ and increasing the size of the microcapsules^18^. The species of animal used is central to the outcome, with a more intense PFO response reported in allogeneic and xenogenic models compared to syngeneic models^19, 20^. The intensity of PFO in rodents is strain specific with a higher fibrotic response seen in C57BL/6 compared to Balb/c mice^21^. A relatively novel strategy to reduce PFO is to co-encapsulate islets with mesenchymal stem cells (MSC). MSC are multipotent and have an important role in tissue repair, promoting angiogenesis and reducing inflammation^22^. They inhibit immune responses by releasing soluble cytokines and growth factors to neighbouring cells^23, 24^. The immunomodulatory properties make them an attractive choice and MSC have been employed in varied studies to improve non-encapsulated islet transplantation outcomes which were attributed to their immunosuppressive effects or enhanced neovascularization^25–29^. A recent study has demonstrated the benefit of co-encapsulating MSC with islets in alginate microcapsules to improve function in a syngeneic transplantation setting using a minimal islet model^30^. The improved graft function in that study was attributed to enhanced insulin secretion yet MSC co-encapsulation provided no benefit in reducing PFO. In another study, MSC co-encapsulation with macroencapsulated pig islets improved graft survival and function by enhancing oxygenation and neoangiogenesis in subcutaneous transplants although any positive MSC effects on the occurrence of PFO was not mentioned^31^.

To date, there are no studies reported in the literature examining the direct effects of MSC co-encapsulation on the occurrence of PFO and encapsulated islet survival in an allotransplantation setting. Further, there are no published studies that examine the effect of co-encapsulating stimulated MSC on PFO, islet survival and function. Stimulating MSC prior to transplantation results in the production of soluble factors which exert a strong immunosuppressive effect compared to unstimulated MSC^32^. In this manuscript, we examined the effect of co-encapsulating both unstimulated and stimulated MSC with islets to test the impact of stimulation on PFO and islet survival. In addition, we investigated the effect of co-transplantation of encapsulated islets and MSC (both unstimulated and stimulated) on PFO and graft survival.

## Results

### Effect of MSC stimulation on cytokine/chemokine secretion

**Figure 1:**
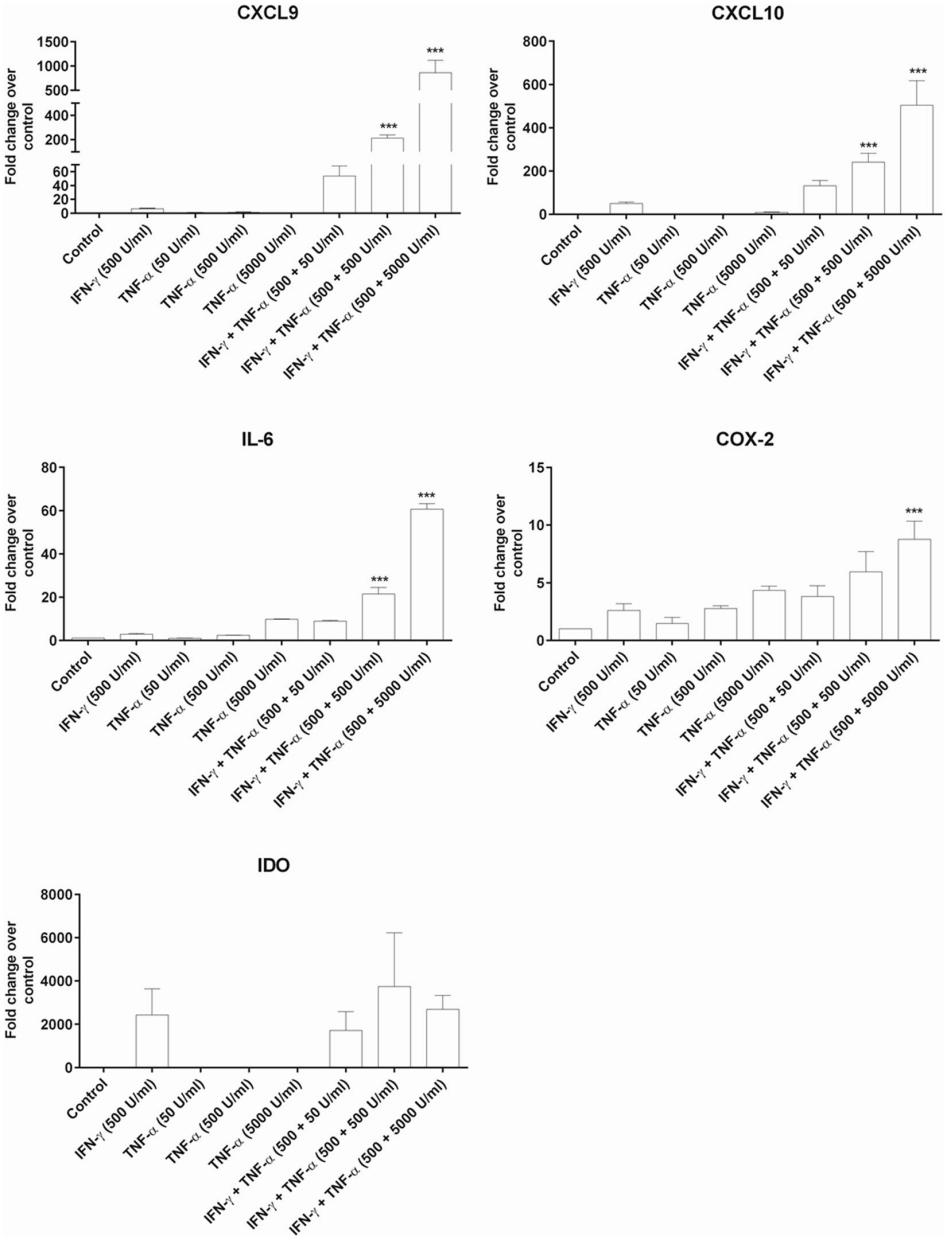
Effect of MSC stimulation on cytokine/chemokine gene expression. MSC stimulation with both IFN-γ+TNF-α (500 + 500 U/mL) and IFN-γ+TNF-α (500 + 5000 U/mL) for 24 h significantly induced the gene expression of chemokines CXCL9 and CXCL10, interleukin IL-6 and enzyme COX-2. MSC stimulation with both IFN-γ+TNF-α (500 + 500 U/mL) and IFN-γ+TNF-α (500 + 5000 U/mL) for 24 h induced IDO gene expression, however the increase was not statistically significant compared to unstimulated MSC. Values = mean ± SEM (n = 3); ***p < 0.0001 for gene fold change of CXCL9, CXCL10 and IL-6 where IFN-γ+TNF-α (500 + 500 U/mL) and IFN-γ+TNF-α (500 + 5000 U/mL) > all other treatment groups and ***p < 0.0001 for gene fold change of COX-2 where IFN-γ+TNF-α (500 + 5000 U/mL) > all other treatment groups (ANOVA with posthoc Duncan’s Multiple Comparison Test).

The multipotency of MSC was confirmed by differentiating them into cells of osteogenic (mineralised deposits that stained with Alizarin red and Von Kossa) and adipogenic lineage (fat droplets stained with Oil Red O) (Supplementary Fig. 1). We next explored the effect of stimulating MSC with proinflammatory cytokines to activate them. Stimulation with IFN-γ (500 U/mL) or TNF-α (50, 500, 5000 U/mL) alone did not significantly induce the gene expression of chemokines (CXCL9 and CXCL10) and the immunomodulatory cytokine (interleukin-6; IL-6) and cyclooxygenase-2 (COX-2) (Fig. 1). Using a cytokine cocktail of interferon-gamma (IFN-γ) and tumour necrosis factor–alpha (TNF-α) at varied concentrations differentially induced the gene expression of CXCL9, CXCL10, IL-6 and COX-2 with a synergistic effect seen by increasing the TNF-α concentration (Fig. 1). In contrast, stimulation with IFN-γ or TNF-α alone or a cocktail of IFN-γ and TNF-α slightly induced indolamine dioxygenase (IDO) gene expression which is known to be expressed in human but not murine MSC^22^, although the induction was not statistically significant compared to unstimulated MSC (Fig. 1). A significant maximal induction of genes CXCL9, CXCL10, IL-6 and COX-2 was seen with the cytokine cocktail IFN-γ+TNF-α (500 + 5000 U/mL) and hence only this cocktail was used for subsequent protein analysis.

**Figure 2:**
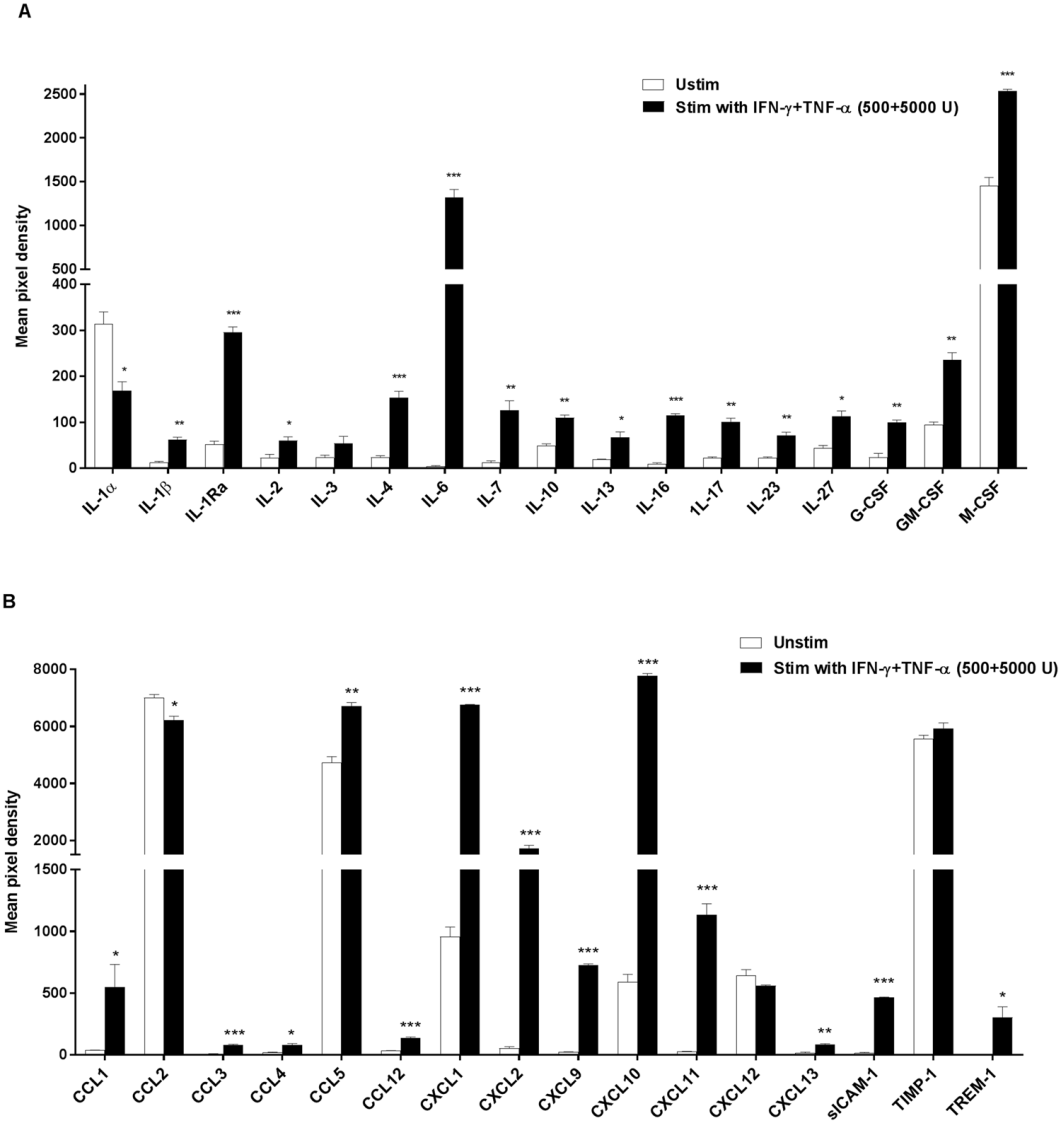
Effect of MSC stimulation on cytokine/chemokine protein expression. MSC stimulation with IFN-γ+TNF-α (500 + 5000 U/mL) significantly induced the secretion of a host of cytokines (**A**) and chemokines (**B**) as measured in the conditioned media 24 h post stimulation. Values = mean ± SEM (n = 2); ***p < 0.0001, **p < 0.001 and *p < 0.05 for mean pixel densities between the unstimulated and stimulated media (Student’s t-test).

A cytokine protein array was carried out to elucidate the cytokine secretion profile of MSC stimulated with IFN-γ+TNF-α (500 + 5000 U/mL). Conditioned media from stimulated MSC had significantly higher levels of cytokines (IL-1β, IL-1Ra, IL-2, −4, −6, −7, −10, −13, −16, −17, −23, −27, G-CSF, GM-CSF, M-CSF), CC chemokines (CCL-1, −3, −4, −5, −12), CXC chemokines (CXCL-1, −2, −9, −10, −11, −13) and other factors (sICAM-1 and TREM-1) compared to unstimulated MSC (Fig. 2A,B). The increased level of proteins CXCL9, CXCL10 and IL-6 seen in the conditioned media of stimulated MSC was consistent with the increased gene expression seen in the quantitative polymerase chain reaction (qPCR) analysis. These data reveal that stimulating MSC with IFN-γ+TNF-α (500 + 5000 U/mL) cocktail for 24 h significantly enhanced the secretion of variety of cytokines/chemokines and most notably the immunomodulatory factors interleukin-1 receptor antagonist (IL-1Ra), interleukin-4 (IL-4), interleukin-6 (IL-6), interleukin-10 (IL-10) and interleukin-13 (IL-13) (Fig. 2A).

### Effect of MSC stimulation on nitric oxide production

**Figure 3:**
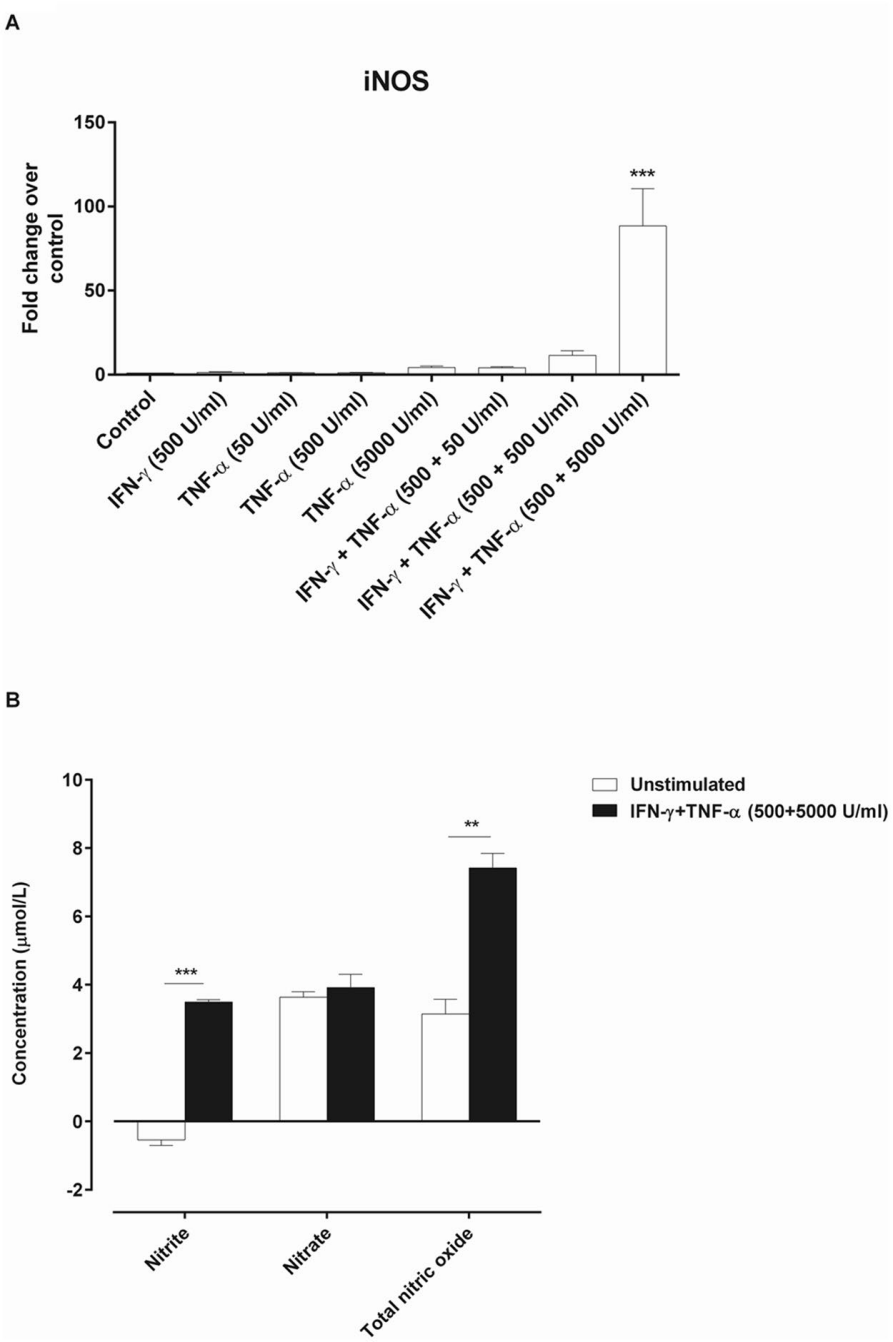
Effect of MSC stimulation on iNOS induction and NO production. MSC stimulation with IFN-γ+TNF-α (500 + 5000 U/mL) for 24 h significantly induced the gene expression of iNOS (**A**). Values = mean ± SEM (n = 3); ***p < 0.0001 for gene fold change of iNOS where IFN-γ+TNF-α (500 + 5000 U/mL) > all other treatment groups (ANOVA with posthoc Duncan’s Multiple Comparison Test). Consistent with gene expression study MSC stimulation with IFN-γ+TNF-α (500 + 5000 U/mL) also significantly enhanced NO production (**B**). Values = mean ± SEM (n = 3); ***p < 0.0001 and **p < 0.01 when compared between unstimulated and stimulated MSC (Student’s t-test).

Previous studies have demonstrated that the murine MSC-mediated immunosuppression largely depends on the nitric oxide (NO) produced by inducible nitric oxide synthase (iNOS)^22^. Accordingly, we examined the effect of MSC stimulation with proinflammatory cytokines on iNOS induction and NO production. Stimulating MSC with IFN-γ (500 U/mL) or TNF-α (50, 500, 5000 U/mL) alone did not significantly induce iNOS gene expression (Fig. 3A). Different to this, MSC stimulation with a cytokine cocktail (IFN-γ and TNF-α) differentially induced iNOS expression in a concentration dependent manner with a significant maximal induction observed with IFN-γ+TNF-α (500 + 5000 U/mL) (Fig. 3A). Consistent with the rise in iNOS gene expression in MSC exposed to IFN-γ+TNF-α (500 + 5000 U/mL) cytokine cocktail, was an increase in total NO production by ~2.4 fold in the medium used for culturing stimulated MSC compared to unstimulated MSC (Fig. 3B).

### Effect of co-encapsulating stimulated MSC on islet viability and function *in vitro*

**Figure 4:**
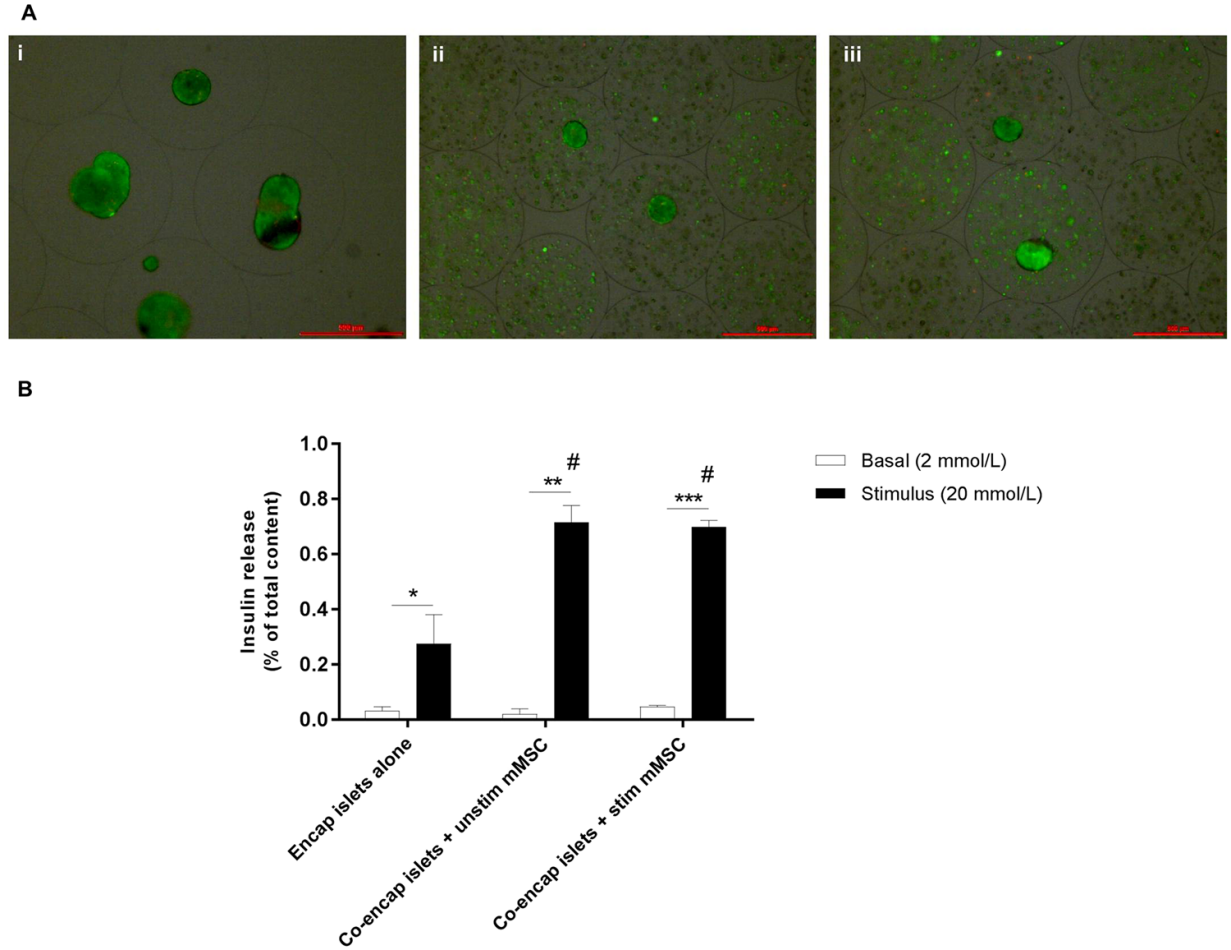
Effect of co-encapsulating stimulated MSC on islet viability and function *in vitro*. Representative viability images of different treatments groups measured at day 1 post-encapsulation (**A**) (Scale bar = 500 μm). Co-encapsulation with either unstimulated (ii) or stimulated (iii) MSC did not affect islet viability compared to encapsulated islets alone (i). Viability of different treatment groups: encapsulated islet alone: 90 ± 1.2%; co-encapsulation with unstimulated MSC: 89.6 ± 1.04% and co-encapsulation with stimulated MSC: 88.7 ± 1.1%. Values = mean ± SEM (n = 3); p > 0.05 (n = 100 islets for each treatment group). Glucose-stimulated insulin secretion of islets co-encapsulated with unstimulated or stimulated MSC compared to encapsulated islets alone measured at day 1 post-encapsulation (**B**). Values = mean ± SEM (n = 3); *p < 0.05, **p < 0.001 and ***p < 0.0001 when compared between basal (2 mmol/L) and stimulus (20 mmol/L) for encapsulated islets alone, co-encapsulated with unstimulated MSC and co-encapsulated with stimulated MSC respectively (Student’s t-test). ^#^p < 0.05 for glucose-induced insulin secretion where stimulus (20 mmol/L) for co-encapsulated islets with either unstimulated or stimulated MSC > stimulus (20 mmol/L) for encapsulated islets alone (ANOVA with posthoc Duncan’s Multiple Comparison Test).

Islets were co-encapsulated with either IFN-γ+TNF-α (500 + 5000 U/mL) stimulated or unstimulated MSC and the viability and insulin secretion assessed. Co-encapsulation with either stimulated or unstimulated MSC did not alter islet viability post-encapsulation (Fig. 4A). Encapsulation did not affect MSC viability and the viability of unstimulated and stimulated MSC were found to be 87.4 ± 0.8% and 86.5 ± 0.7% respectively. Co-encapsulation of islets with unstimulated and stimulated MSC significantly increased glucose-stimulated insulin secretion by ~2.6 and ~2.5 fold respectively compared to encapsulated islets, with no effect on basal insulin secretion (Fig. 4B).

### Minimal islet mass needed to normalize blood glucose levels in diabetic C57BL/6 mice

To determine the minimal islet mass, encapsulated islets (1000 or 500 IEQ) were transplanted into the peritoneal cavity of diabetic immunocompetent C57 black 6 (C57BL/6) and the outcomes compared against those transplanted into diabetic immunodeficient non-obese diabetic/severe combined immunodeficient (NOD/SCID) mice. Blood glucose levels (BGLs) of C57BL/6 mice allotransplanted with either 1000 or 500 IEQ declined from 25.03 ± 1.3 to 9.5 ± 0.6 and became normoglycemic by day 5 post-transplantation (Supplementary Fig. 2A). However, BGLs started to rise progressively in both the groups after day 12 and the percentage of normoglycemic mice declined significantly to 0% and 20% for 500 and 1000 IEQ respectively at the day 24 end-point (Supplementary Fig. 2B). To assess graft function, an intraperitoneal glucose tolerance test (IPGTT) was performed at day 24 post-transplantation. Mice transplanted with either 500 or 1000 IEQ had an abnormal glucose clearance value which was significantly lower than the non-diabetic controls (Supplementary Fig. 2C). In contrast, transplantation of either 1000 or 500 IEQ into immunodeficient NOD/SCID mice normalized BGLs and all mice remained normoglycemic until the end of study period at day 62 post-transplantation, with a normal glucose tolerance test (Supplementary Fig. 3A,B).

**Figure 5:**
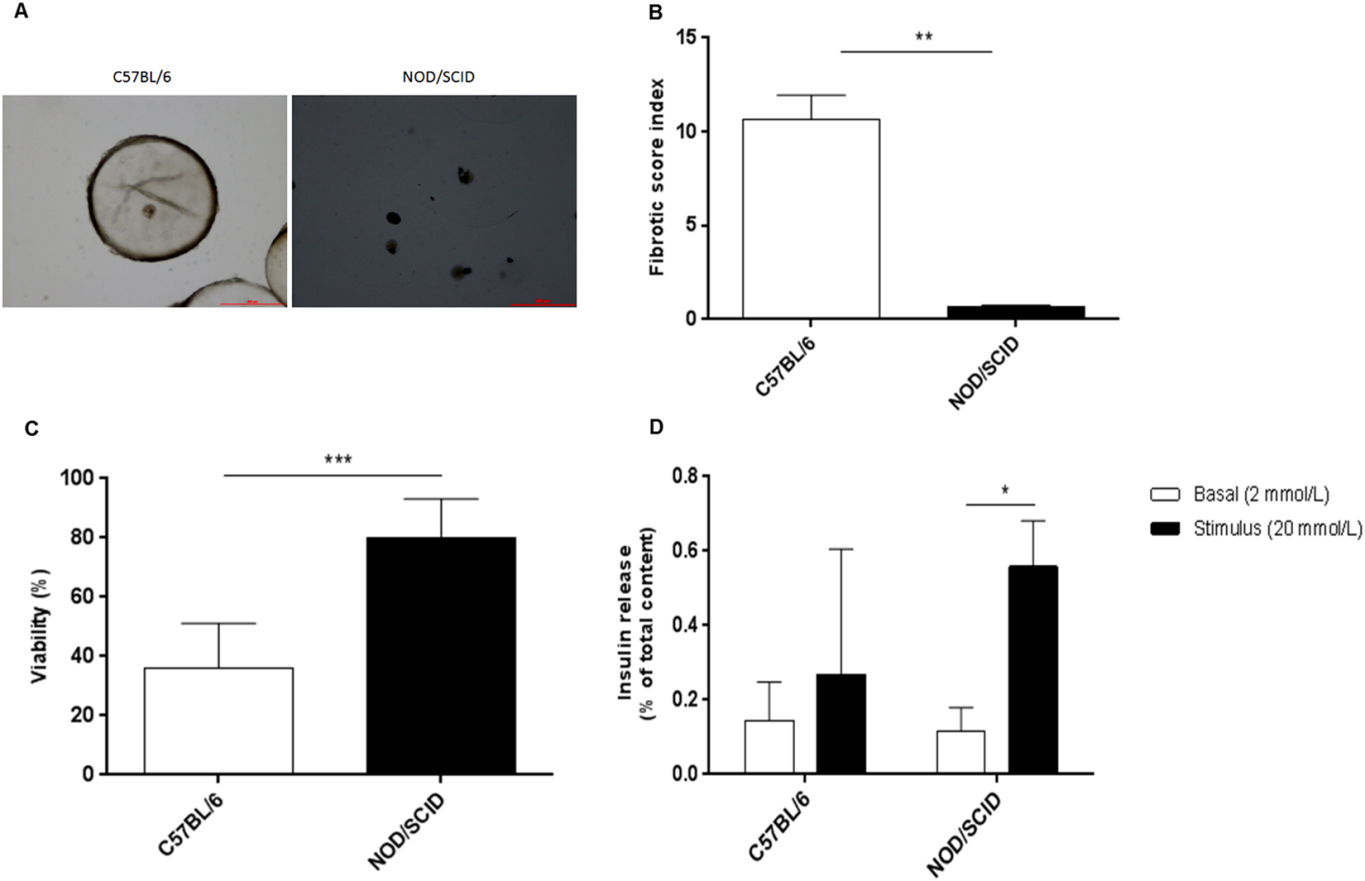
Assessment of grafts retrieved from C57BL/6 and NOD/SCID mice transplanted intraperitoneally with 500 IEQ. Representative images of encapsulated islets retrieved from C57BL/6 and NOD/SCID mice at 24 and 62 days respectively post-transplantation (**A**) (Scale bar = 500 μm). The degree of PFO measured as fibrotic score index on grafts retrieved from C57BL/6 and NOD/SCID mice transplanted (**B**); Values = mean ± SEM (n = 5 for C57BL/6 and n = 3 for NOD/SCID); **p < 0.01 for fibrotic score index between C57BL/6 and NOD/SCID (Student’s t-test). Viability of encapsulated islets retrieved from C57BL/6 and NOD/SCID mice (**C**); Values = mean ± SEM (n = 100 islets for each mouse strain); ***p < 0.0001 for viability between C57BL/6 and NOD/SCID (Student’s t-test). *Ex-vivo* static stimulation studies on grafts retrieved from C57BL/6 and NOD/SCID mice (**D**); Values = mean ± SEM (n = 5 for C57BL/6 and n = 3 for NOD/SCID); *p < 0.05 when compared between basal (2 mmol/L) and stimulus (20 mmol/L) for grafts retrieved from NOD/SCID (Student’s t-test).

Microcapsules retrieved from C57BL/6 showed the presence of dense PFO (Fig. 5A) with a fibrotic score index of 10.6 ± 1.3 compared to little or no fibrotic overgrowth on those retrieved from NOD/SCID (fibrotic score index of 0.7 ± 0.07) (Fig. 5B). The presence of dense PFO in the C57BL/6 group significantly reduced islet survival with viability of 35.9 ± 2.2% compared to islet viability of 80.1 ± 1.3% in the NOD/SCID group (Fig. 5C). A similar outcome was seen in the *ex vivo* static stimulation studies with grafts retrieved from C57BL/6 responding poorly to a glucose challenge compared to those retrieved from NOD/SCID (Fig. 5D). Together, data demonstrated that the presence of PFO was a major factor impeding graft survival and function. This study also showed that a minimal mass of 500 IEQ of encapsulated islets was unable to normalise blood glucose levels when allotransplanted, an outcome we attributed to a strong PFO response adversely affecting graft survival and function. For this reason, we used this minimal islet C57BL/6 model to determine the effects of stimulated or unstimulated MSC on PFO, islet survival and function in all subsequent studies.

### Effect of co-encapsulating or co-transplanting stimulated MSC on PFO, islet survival and function *in vivo*

**Figure 6:**
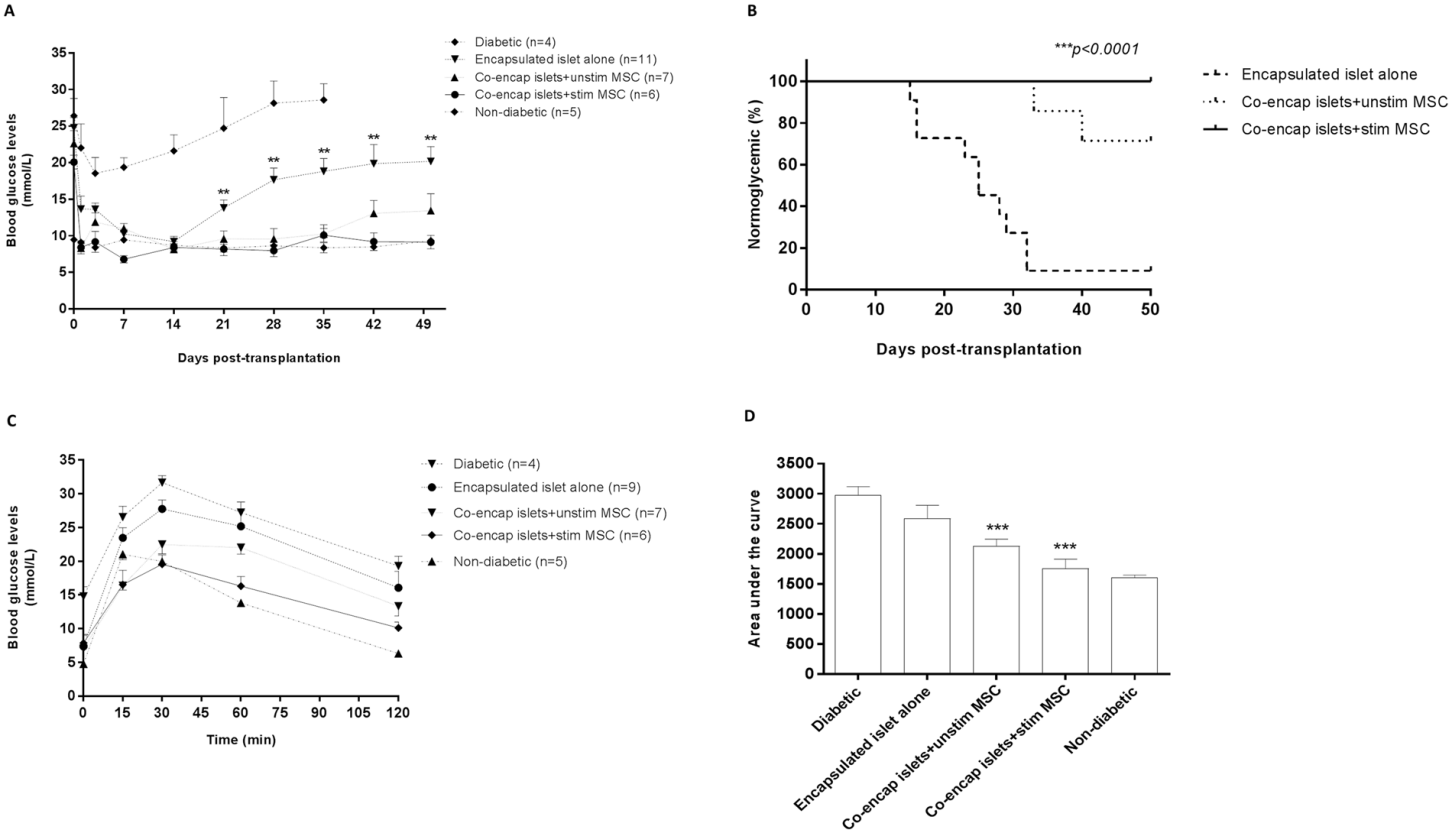
Transplantation of islets co-encapsulated with stimulated or unstimulated MSC into diabetic C57BL/6 mice. Random average blood glucose levels of diabetic C57BL/6 mice allotransplanted intraperitoneally with 500 IEQ of encapsulated islets co-encapsulated with stimulated or unstimulated MSC (**A**); Values = mean ± SEM; **p < 0.01 for BGLs at days 21, 28, 35, 42 and 50 where encapsulated islets alone > co-encapsulated islet with unstimulated or stimulated MSC (ANOVA with posthoc Duncan’s Multiple Comparison Test). Percentage of mice that became normoglycemic when transplanted with 500 IEQ of encapsulated islets co-encapsulated with stimulated or unstimulated MSC (**B**); ***p < 0.0001 (Kaplan-Meier survival analysis [log-rank]). IPGTT carried out at day 49 post-transplantation (**C**); Values = mean ± SEM. Area under the curve (AUC) for IPGTT values (**D**); Values = mean ± SEM; ***p < 0.0001 for AUC where co-encapsulated islets + stimulated MSC < diabetic controls and encapsulated islets alone; and ***p < 0.0001 for AUC where co-encapsulated islets + unstimulated MSC < diabetic controls and encapsulated islets alone; and co-encapsulated islets + unstimulated MSC > non-diabetic controls. There is no significant difference for AUC between co-encapsulated islets + stimulated MSC and non-diabetic controls (ANOVA with posthoc Duncan’s Multiple Comparison Test).

Mouse islets were co-encapsulated with unstimulated or stimulated MSC within the same capsule (at a ratio of 1:1) and allotransplanted into the peritoneal cavity of C57BL/6 mice. Co-encapsulation of islets with stimulated MSC improved the graft outcome significantly compared to unstimulated MSC and encapsulated islets alone. All the mice receiving stimulated MSC became normoglycemic earlier, at 2 ± 1 days (range 1–7 days) post-transplantation, compared to those receiving unstimulated MSC and encapsulated islets alone which became normoglycemic by 5.4 ± 0.8 (range 1–7 days) and 5.4 ± 1.8 (range 1–14 days) days respectively (Fig. 6A). The average BGLs for the stimulated MSC group were significantly lower, on days 21, 28, 35, 42 and 50 respectively, compared to encapsulated islets alone. At day 50 post-transplantation, 100% of the mice in the group transplanted with stimulated MSC remained normoglycemic compared to 71.4% in the unstimulated MSC group. Only 9.1% of the mice in the control encapsulated islet alone group were normoglycemic (Fig. 6B).

**Figure 7:**
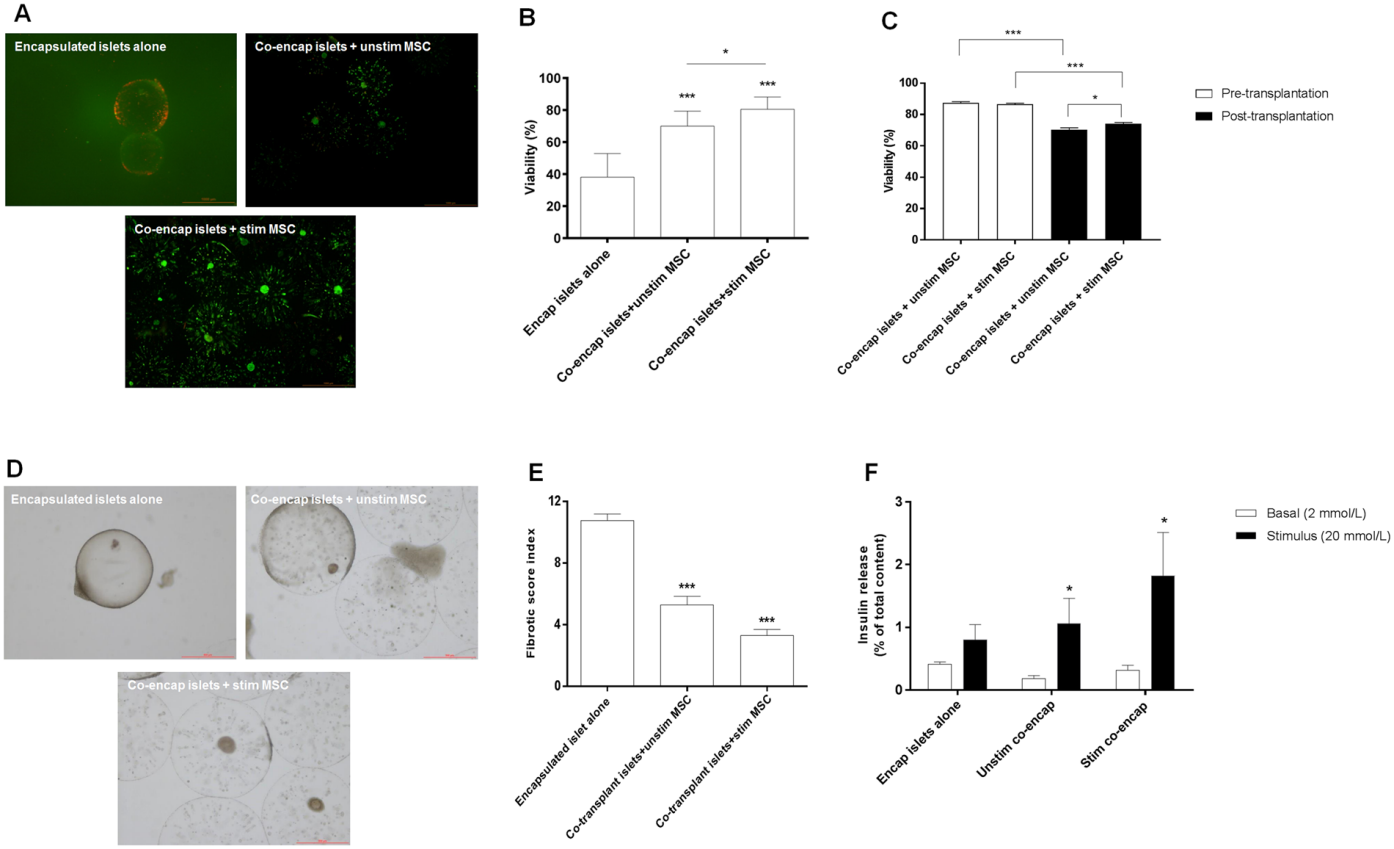
Assessment of grafts retrieved from C57BL/6 transplanted with islets co-encapsulated with stimulated or unstimulated MSC. Representative viability images of grafts retrieved from different treatment groups at day 50 post-transplantation (**A**) (Scale bar = 1000 μm). Percentage viability of encapsulated islets retrieved from different treatment groups at day 50 post-transplantation (**B**). Values = mean ± SEM (n = 100 islets for each treatment group); ***p < 0.0001 for viability where co-encapsulated islets + unstimulated MSC and co-encapsulated islets + stimulated MSC > encapsulated islets alone (ANOVA with posthoc Duncan’s Multiple Comparison Test) and *p < 0.05 for viability when compared between co-encapsulated islets + unstimulated MSC and co-encapsulated islets + stimulated MSC (Student’s t-test). Percentage viability of encapsulated MSC retrieved from different treatment groups at day 50 post-transplantation (**C**). Viability of co-encapsulated MSC pre-transplantation (unstimulated vs stimulated: 87.4 ± 0.8 vs 86.5 ± 0.7%) and at day 50 post-transplantation (unstimulated vs stimulated: 70.3 ± 1.2 vs 74.02 ± 0.9%). Values = mean ± SEM (n = 100 encapsulated MSC for each treatment group); ***p < 0.001 for viabilities of both unstimulated and stimulated MSC where viability of pre-transplanted group > post-transplanted group and *p = 0.0378 for viability between unstimulated and stimulated MSC groups at day 50 post-transplantation (ANOVA with posthoc Duncan’s Multiple Comparison Test). Representative images of grafts retrieved from different treatment groups showing the degree of PFO at 50 days post-transplantation (**D**) (Scale bar = 500 μm). Extent of PFO on retrieved grafts represented as fibrotic score index, on a scale of 0 (no fibrotic overgrowth) to 16 (complete fibrotic overgrowth) (**E**); Values = mean ± SEM (n = 6–9); ***p < 0.0001 for fibrotic score index where encapsulated islets alone > co-encapsulated islets with unstimulated or stimulated MSC (ANOVA with posthoc Duncan’s Multiple Comparison Test). *Ex-vivo* static stimulation studies on grafts retrieved from different treatment groups (**F**); Values = mean ± SEM (n = 5–7); *p < 0.05 when compared between basal (2 mmol/L) and stimulus (20 mmol/L) for grafts retrieved from co-encapsulated groups containing either unstimulated or stimulated MSC (Student’s t-test).

To further assess graft function, an IPGTT was carried out at day 49 post-transplantation. Animals transplanted with stimulated MSC handled glucose normally and had lower blood glucose levels (and areas under the curve; AUC) compared to mice that received unstimulated MSC or encapsulated islets alone (Fig. 6C,D). There was no significant difference in the area under the curve (AUC) between unstimulated and stimulated MSC. However, AUC for unstimulated MSC was significantly higher compared to non-diabetic controls as opposed to AUC for stimulated MSC being similar to non-diabetic controls (Fig. 6D). The viability of islets retrieved from the stimulated MSC group at 80.6 ± 2.6% was significantly higher than that for the unstimulated MSC group at 70 ± 1.8% (p < 0.05). This contrasted to the viability of islets encapsulated alone that was significantly reduced at 38.1 ± 2.1% (Fig. 7A,B). Similarly, the viability of stimulated MSC at day 50 post-transplantation was higher compared to unstimulated MSC, however their viabilities were significantly less compared to pre-transplantation values (Fig. 7C and Supplementary Fig. 6). Assessment of PFO showed that grafts retrieved from groups receiving either stimulated or unstimulated MSC had significantly less fibrotic overgrowth (Fig. 7D) with a fibrotic score index of 1.7 ± 0.5 and 4.2 ± 1.03 respectively compared to encapsulated islets alone which had a very high fibrotic score index of 10.7 ± 0.4 (Fig. 7E). Further, the level of PFO in the stimulated MSC group was less compared to unstimulated MSC group again suggesting a benefit of stimulating MSC prior to transplantation. *Ex vivo* static stimulation studies on retrieved grafts showed that glucose-stimulated insulin secretion was higher for islets co-encapsulated with either stimulated or unstimulated MSC compared to encapsulated islets alone (Fig. 7F).

A similar outcome was obtained to that of the co-encapsulation study when encapsulated islets and stimulated/unstimulated MSC (1:1 ratio) in separate capsules were co-transplanted into the peritoneal cavity of diabetic C57BL/6 mice (Supplementary Figs 4 and 5).

### Evaluation of peritoneal cytokines/chemokines

**Figure 8:**
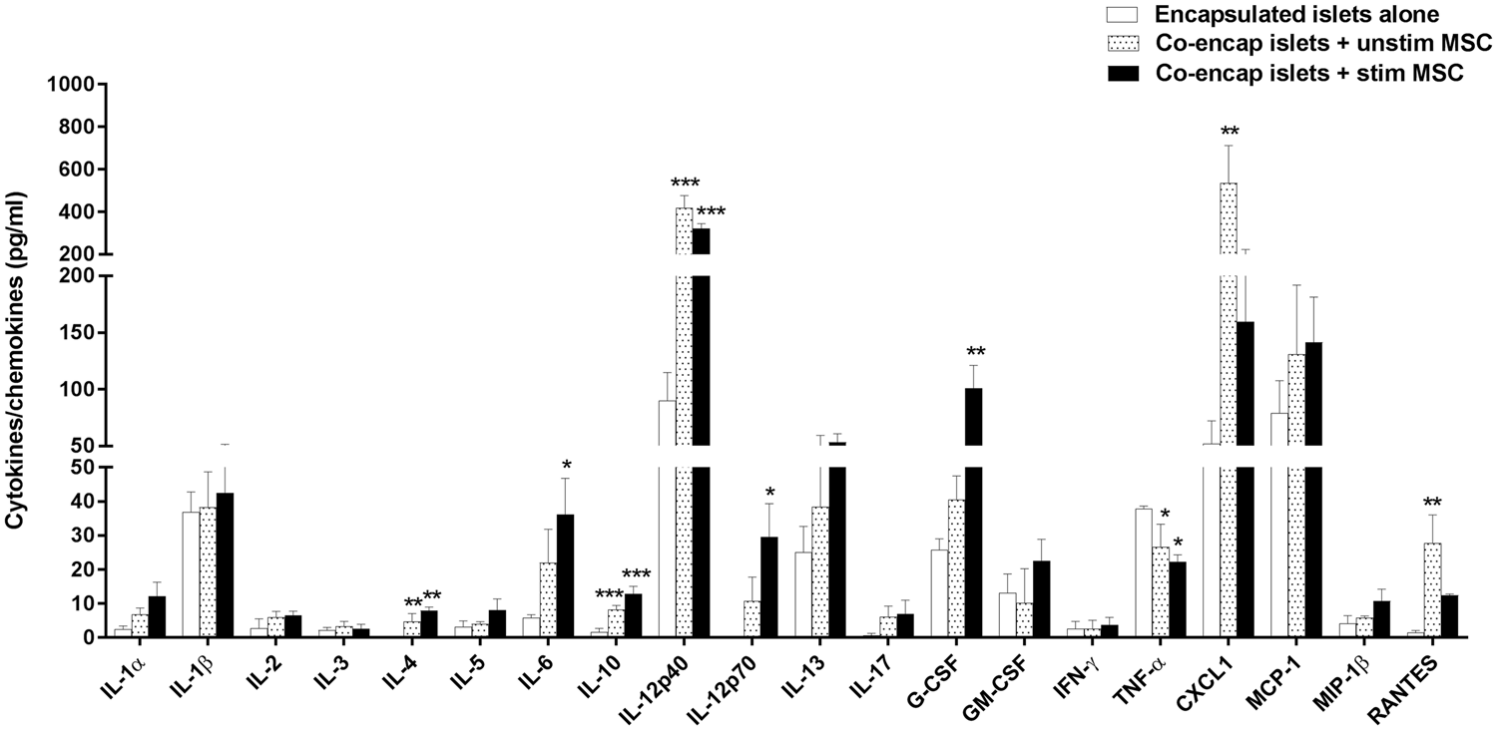
Analysis of peritoneal cytokines/chemokines. Levels of cytokines/chemokines measured in the peritoneal fluid of C57BL/6 mice transplanted with islets co-encapsulated with stimulated or unstimulated MSC at day 50 post-transplantaion. Values = mean ± SEM (n = 4–5); *p < 0.05, **p < 0.01 and ***p < 0.001 for cytokines/chemokines levels where islets co-encapsulated with unstimulated or stimulated MSC > encapsulated islets alone (ANOVA with posthoc Duncan’s Multiple Comparison Test).

Analysing the peritoneal cytokines/chemokines increased our understanding of the immune response occurring in the transplants. Comparing the co-encapsulated groups, there was no significant difference in the levels of cytokines (IL-1α, IL-1β, IL-2, IL-3, IL-5, IL-13, IL-17, GM-CSF, IFN-γ) and chemokines (MCP-1, MIP-1β) in all the three groups measured at day 50 post-transplantation (Fig. 8). On the other hand, the levels of cytokines interleukin-12p40 (IL-12p40) and interleukin-12p70 (IL-12p70) and chemokines C-X-C ligand 1 (CXCL1) and Regulated on activation, normal T cell expressed and secreted (RANTES) were higher in the MSC co-encapsulated groups compared to encapsulated islets alone. Of the varied cytokines measured, the most interesting are the immunomodulatory cytokines IL-4, IL-6, IL-10 and granulocyte-colony stimulating factor (G-CSF). Levels of IL-4 and IL-10 were significantly higher in both the unstimulated and stimulated MSC groups compared to encapsulated islets alone. However, levels of IL-6 and G-CSF were significantly higher only in the stimulated MSC group compared to unstimulated MSC and encapsulated islets alone (Fig. 8). On the contrary, TNF-α levels were significantly reduced in both MSC co-encapsulated groups compared to islets encapsulated alone. Comparing the co-transplanted group, the cytokine and chemokine expression followed a similar pattern to the co-encapsulated group except that IL-4 was not detectable in the MSC co-transplanted groups (Supplementary Fig. 7). Levels of IL-9, macrophage inflammatory protein-1 alpha (MIP-1α) and eotaxin were below the detection limit of the assay and hence could not be detected.

## Discussion

Bone marrow derived mesenchymal stem cells (MSC) are highly efficient in suppressing autoimmunity, graft rejection and other immune disorders both in preclinical and clinical settings^33–37^. However, this immunomodulatory ability of MSC is not intrinsic, rather induced by proinflammatory cytokines such as IFN-γ in combination with TNF-α or IL-1β^38^. Exposing MSC to inflammatory signals significantly enhances their immunosuppressive effects on T-cells, macrophages and dendritic cells^32, 39–41^. We hypothesized that stimulating MSC prior to transplantation would enhance their immunomodulatory effects and hence improve the survival and function of encapsulated islets when allografted. Our findings showed that IFN-γ in combination with TNF-α synergistically enhanced the immunosuppressive effects of murine MSC. They induced iNOS and enhanced NO production, a potent immunosuppressor which plays a major role in modulating the T cell immune response^42, 43^. However, NO produced has a short half-life and can only act locally, so the immune cells need to be in close proximity to MSC suggesting the importance of chemotaxis in NO-mediated immunosuppression. In this study, stimulating MSC upregulated the expression of chemokines belonging to both CC and CXC families especially CXCL-9, −10 and −11 that were T-cell specific chemoattractants^44^, suggesting that stimulated MSC attract T cells and when in proximity can exert an immunosuppressive effect on them mediated by NO. Additionally, stimulated MSC secreted a wide range of pro-inflammatory and anti-inflammatory cytokines with significantly higher levels of immunomodulatory cytokines IL-1ra, IL-4, IL-6, IL-10 and IL-13 compared to unstimulated MSC. These immunomodulatory cytokines play a major role in suppressing inflammatory responses and shifting the immune balance towards an anti-inflammatory phenotype^45^.

The choice of animal model was crucial in this study as the severity of PFO is highly variable in murine strains^21^. We chose C57BL/6 as the immune response in this strain is vigorous and produces a fibrotic response that is similar to that seen in humans^46^. Using diabetic C57BL/6 as recipients, we found that 500 IEQ of encapsulated Quackenbush Swiss (QS) islets failed to normalize BGLs in 100% of the mice. This result was consistent with another study where 500 IEQ of encapsulated mouse (DBA/2) islets failed to normalize BGLs when allotransplanted under the kidney capsules of C57BL/6 mice^47^. The graft failure seen in the allotransplanted C57BL/6 model is largely attributed to PFO.

The beneficial effect of MSC on islet survival and function has been demonstrated previously in co-culture studies where MSC improved viability, increased insulin secretion and content and reduced apoptosis^48–51^. Another study demonstrated a benefit where islets co-encapsulated with MSC significantly increased stimulatory insulin secretion as well as insulin content^30^. Similarly, our study showed that co-encapsulation of islets with MSC enhanced glucose induced insulin secretion *in vitro* without altering viability. The benefit observed is possibly due to the insulinotropic effect of NO released by the MSC^52^. *In vivo* studies also have demonstrated the benefit of transplanting non-encapsulated islets with MSC including prolonging islet survival, enhancing angiogenesis and reducing the number of islets needed to achieve normoglycemia^25, 26, 28, 53^. However, none of these studies investigated the effect of stimulated MSC on islet survival and function. Another study demonstrated that co-encapsulation of islets with unstimulated MSC improved graft survival and normalized blood glucose levels in 71% C57BL/6 mice syngeneically transplanted^30^. However, in that study unstimulated MSC had no effect on PFO when scored at 6 weeks post-transplantation^30^. This contrasted to findings in our allogeneic study where co-encapsulation with both unstimulated and stimulated MSC significantly reduced PFO compared to encapsulated islets alone. There is no significant difference in the PFO reduction with or without MSC stimulation, despite stimulated MSC having a lower fibrotic score index. However, such a slightly increased reduction in PFO seen with the stimulated MSC is sufficient to significantly enhance islet viability and graft survival with normoglycemia being achieved in 100% mice as opposed to 71.4% seen with unstimulated MSC. PFO consisting mostly of macrophages and fibroblasts can secrete toxic chemokines/cytokines which can enter the pores of the microcapsules and have deleterious effects on islets contained inside. A similar outcome was obtained in our co-transplantation study with stimulated MSC providing better efficacy than unstimulated MSC. Our data suggest that the benefit observed by co-encapsulating or co-transplanting MSC with the allografted islets is wholly or partly due to their ability to reduce PFO, since there is a correlation between degree of reduction in PFO and the metabolic outcome. It cannot be due to enhanced angiogenesis because blood vessels do not enter the microcapsules.

The reduction in PFO seen with both co-encapsulated and co-transplanted MSC might be attributed to the secretion of immunomodulatory cytokines that are known to modulate the immune response. Mice transplanted with co-encapsulated or co-transplanted MSC had higher levels of the immunomodulatory cytokines IL-4, IL-6, IL-10, and G-CSF compared to encapsulated islets alone. Moreover, the level of these immunomodulatory cytokines was higher in the stimulated MSC group compared to those receiving unstimulated MSC though not significant. These immunomodulatory cytokines can easily permeate through the microcapsule pores with a molecular weight cut-off of ~250 kDa^54^ and enter the host system to modulate the immune response. Of these, IL-10 possess strong immunosuppressive properties and IL-10 induction has been shown to prolong islet allograft survival^55, 56^. IL-4 inhibit macrophage activation^45^ and IL-6 inhibit T-cell proliferation and protects beta cells from the deleterious effects of proinflammatory cytokines^57, 58^. G-CSF has been known to modulate the immune response and inhibit proinflammatory cytokine production^59^. On the contrary, there was no difference in the levels of most proinflammatory cytokines IL-1β, IL-2 and IFN-γ among the groups transplanted, with the exception of TNF-α. Its reduced level in both co-encapsulated and co-transplanted MSC groups might be attributed to upregulation of IL-10 and G-CSF, which has direct inhibitory effects on TNF-α production^60, 61^.

In conclusion, our results demonstrate that stimulating MSC with proinflammatory cytokines enhanced their immunosuppressive potential by secreting various immunomodulatory cytokines and nitric oxide. We also show that both co-encapsulation and co-transplantation of islets with MSC is a useful strategy to reduce PFO and improve encapsulated islet allograft survival and function with stimulated MSC providing a slightly better outcome than unstimulated MSC. We attribute this benefit to the upregulation of anti-inflammatory cytokines IL-4, IL-6, IL-10 and G-CSF, which are known to modulate the immune response. Prior to clinical translation into people with type 1 diabetes, studies should be carried out with stimulated human MSC co-encapsulated with human islets and tested in an allotransplantation setting using a humanized mice model.

## Methods

### Animals

Animal usage and all experimental procedures were approved by the “Animal Care and Ethics Committee” of Commonwealth scientific and Industrial Research Organization (CSIRO). All experiments with animals were performed in accordance with the CSIRO guidelines and regulations. All animals were sourced from the Australian Resource Centre (Canning Vale, Western Australia). Male Quackenbush Swiss (QS) mice (10–12 weeks) were used as islet donors, and recipients were female immunocompetent C57BL/6 mice (10–12 weeks) and female non-obese diabetic/severe combined immunodeficient (NOD/SCID) mice (8–10 weeks).

### Islet isolation

Islets were isolated from male QS mice (10–12 weeks) as described previously^62^ and the protocol is described in the Supplementary Methods. The purity of the isolated islets was assessed by dithizone (Sigma, St Louis, MO) staining and found to be >95%.

### MSC stimulation

The protocol for the culture and differentiation of MSC into osteogenic or adipogenic lineages were described in the Supplementary Methods. Briefly, MSC were plated onto 6 well plates at a concentration of 1 × 10^4^ cells/cm^2^ and adhered for 2 days. After that the cells were washed twice with phosphate buffered saline and incubated in culture medium with or without stimulating agents for 24 h with proinflammatory cytokines interferon-gamma (IFN-γ; 500 U/mL) (R&D Systems, Minneapolis, MN) and tumour necrosis factor-alpha (TNF-α; 50, 500 and 5000 U/mL) (R&D Systems) alone or by a proinflammatory cytokine cocktail of IFN-γ (500 U/mL) and TNF-α at varied concentrations of 50, 500 and 5000 U/mL.

### Gene expression

Real-time qPCR was performed to determine the gene expression levels of CXCL9, CXCL10, IL-6, cyclooxygenase-2 (COX-2), indoleamine 2,3 di-oxygenase (IDO) and inducible nitric oxide synthase (iNOS). The primer sequences used are listed in Table 1 and protocol is described in the Supplementary Methods.

**Table 1:** Primer sequences for the varied genes analysed in real-time qPCR.

Gene	Forward primer	Reverse primer
IDO	5′-ACG GGC AGC TTCGAGAAG -3′	5′-TCG CAG TAG GGA ACA GCA A -3′
iNOS	5′-CGAAACGCTTCACTTCCA A-3′	5′-TGAGCCTATATTGCTGTGGCT-3′
COX-2	5′-AACCGCATTGCCTCTGAAT-3′	5′-CATGTTCCAGGAGGATGGAG-3′
CXCL9	5′-CTTTTCCTCTTGGGCATCAT-3′	5′-GCATCGTGCATTCCTTATCA-3′
CXCL10	5′-GCTGCCGTCATTTTCTGC-3′	5′-TCTCACTGGCCCGTCA-3′
IL-6	5′-CAAAGCCAGAGTCCTTCAGAG-3′	5′-GCCACTCCTTCTGTGACTCC-3′
RPL13A	5′-ATGACAAGAAAAAGCGGATG-3′	5′-CTTTTCTGCCTGTTTCCGTA-3′

### Nitric oxide assay

2 × 10^5^ MSC were cultured with/without a cocktail of proinflammatory cytokines IFN-γ+TNF-α (500 + 5000 U/mL) for 24 h and culture supernatants collected for measurement of nitric oxide by colorimetry using a nitrite/nitrate (NO_2_/NO_3_) kit obtained from Enzo Life Sciences, Farmingdale, NY.

### Mouse cytokine array profiler

2 × 10^5^ MSC were cultured with/without a cocktail of proinflammatory cytokines IFN-γ+TNF-α (500 + 5000 U/mL) for 24 h and culture supernatants collected for cytokine/chemokine analysis using a dot blot mouse cytokine array panel as per manufacturer’s instructions (R&D Systems).

### Microencapsulation

The number of MSC required for microencapsulation was calculated on the basis of 1 IEQ has ~1500 cells, as described previously^63^. Accordingly, the islets and MSC were used in a ratio of 1:1 (for 500 IEQ the number of MSC required was 7.5 × 10^5^) for the co-encapsulation (islets and MSC in the same capsule) and co-transplantation (islets and MSC in separate capsules) experiments. The cells were encapsulated within barium alginate microcapsules using a air driven droplet generator and the viability of encapsulated islets assessed using the fluorescent dyes 6-carboxy fluorescein diacetate (6-CFDA) and propidium iodide (PI). The ability of co-encapsulated or co-transplanted islets to secrete insulin was assessed in static stimulation studies by exposing to either 2.8 mM glucose (basal) or 20 mM glucose (stimulus). A detailed protocol on the microencapsulation procedure, viability assessment and static stimulation studies are described in the Supplementary Methods.

### Transplantation & glucose tolerance tests

Recipient female immunocompetent C57BL/6 mice (10–12 weeks) and female immunodeficient NOD/SCID mice (8–10 weeks) were made diabetic by intraperitoneal injection of streptozotocin (STZ) (Sigma) at 50 mg/kg (for 5 consecutive days on animals fasted overnight) and 70 mg/kg (for 3 consecutive days) respectively 1–2 weeks prior to transplantation. Diabetic recipient C57BL/6 and NOD/SCID mice were transplanted intraperitoneal with either 500 or 1000 IEQ to determine the minimal islet mass. Once the minimal islet mass was determined, subsequent experiments were carried out in diabetic C57BL/6 mice with unstimulated/stimulated MSC either co-encapsulated or co-transplanted with islets to determine graft survival and function. Animals were considered normoglycemic if three consecutive blood sugar levels were <11.1 mmol/L. A detailed protocol on diabetes induction, transplantation procedure, different transplant groups and glucose tolerance tests is described in the Supplementary Methods.

### Graft retrieval & assessment of PFO

At the end of the study period, on day 50 post-transplantation, microcapsules were retrieved by peritoneal lavage (using a minimal volume of 2 ml) and assessed for PFO, viability, insulin secretion and intraperitoneal cytokines as described previously^64^, a detailed procedure of which is provided in the Supplementary Methods. Microcapsules were observed under the microscope and degree of PFO was assessed in a blinded fashion using a scoring system. Briefly, the microcapsules were scored as follows: 0 = no overgrowth, 1 = < 25% of microcapsule overgrown, 2 = 25–50% of microcapsule overgrown, 3 = 50–75% of microcapsule overgrown and 4 = > 75% of microcapsule overgrown. The fibrotic score index was calculated for each mouse using the formula (0 × % score 0) + (2 × % score 1) + (4 × % score 2) + (8 × % score 3) + (16 × % score 4), giving a minimum possible fibrotic score of 0 and a maximum possible fibrotic score of 16.

### Assessment of peritoneal cytokines/chemokines

Quantification of different cytokines and chemokines in the peritoneal fluid was measured with Luminex technology using a Bioplex mouse cytokine 23-plex cytokine kit (Bio-Rad Laboratories, Hercules, CA), according to the manufacturer’s protocol at day 50 post-transplantation.

### Statistical analysis

All data are presented as mean ± standard error of mean (SEM). Differences between two groups were analyzed by the two-tailed Student’s t-test and more than two groups by one-way analysis of variance (ANOVA) with *post-hoc* Duncan’s Multiple-Comparison test using NCSS 2004 (NCSS, Kaysville, UT). For the survival curves, the Kaplan-Meier survival analysis log-rank test was used. Significant differences among data groups were assigned when p < 0.05.

## Electronic supplementary material

Supplementary Methods

Dataset 1
