# Supplementary material for: Co-encapsulation and co-transplantation of mesenchymal stem cells reduces pericapsular fibrosis and improves encapsulated islet survival and function when allografted
Source: Sci Rep. 2017 Aug 30;7:10059. doi: 10.1038/s41598-017-10359-1 (PMC5577272; doi:10.1038/s41598-017-10359-1)
Supplement: Supplementary file 1 — Supplementary Methods [file 41598_2017_10359_MOESM1_ESM.doc]

**Co-encapsulation and co-transplantation of mesenchymal stem cells reduces pericapsular fibrosis and improves encapsulated islet survival and function when allografted**

Vijayaganapathy Vaithilingam, PhD^1,*^, Margaret DM Evans, PhD^1^, Denise M Lewy, BSc^1^, Penelope A Bean, BSc^1^, Sumeet Bal, MSc^1^ & Bernard E Tuch, MD, PhD^1,2^

^1^Biomedical Manufacturing Research Program, Commonwealth Scientific and Industrial Research Organization (CSIRO), Manufacturing Flagship, North Ryde, New South Wales, Australia.

^2^Australian Foundation for Diabetes Research, Sydney, New South Wales, Australia, previously at CSIRO Manufacturing Flagship, North Ryde, New South Wales, Australia.

**Supplementary Methods**

**Islet isolation.** Pancreatic islets from male QS mice (10-12 weeks) were isolated by ductal perfusion of collagenase (Liberase RI, Roche, NSW, Australia), followed by purification on Ficoll-Paque gradients (GE Healthcare, Chalfont St Giles, UK) and separated from unwanted tissue debris using a stereomicroscope. The isolated islets were then cultured for 48 h in RPMI 1640 medium (Life Technologies, Melbourne, Australia) supplemented with penicillin and streptomycin and 10% foetal bovine serum (FBS) (Bovogen Biologicals, Melbourne, Australia) in an incubator at 37 ^o^C supplied with 5% CO_2_ in air before being encapsulated. The purity of the islets was assessed by dithizone (Sigma, St Louis, MO) staining.

**MSC.** Mouse mesenchymal stem cells (MSC) isolated from C57BL/6 bone marrow were purchased from Invitrogen (Melbourne, Australia) and expanded in Dulbecco's **Modified Eagle Medium/Nutrient Mixture** (DMEM/F-12) (Life Technologies) with penicillin/streptomycin and 10% FBS. MSC exhibit a population doubling time of ~21 to 23 hours and care was taken only to use MSC with low passages (below 10 passages) as MSC above passage 11 tend to lose their potential. The multipotency of MSC was tested (at same passage as those used in animal experiments) by culturing them in either an osteogenic or adipogenic differentiation media. MSC in the osteogenic conditions were cultured in alpha-Minimum Essential Media α-MEM (Life Technologies) supplemented with penicillin/streptomycin, dexamethasone (40 ng/mL), glycerol-2-phosphate (1500 µg/mL) and ascorbic acid (50 µg/mL) with 10% FBS for 28 days and then routinely stained with Alizarin Red (Sigma) and Von Kossa stain using silver nitrate (Sigma) to identify mineralised deposits which occur when cells differentiate into osteocytes. MSC in adipogenic conditions were cultured in DMEM/F-12 (Life Technologies) supplemented with pencillin/streptomycin, dexamethasone (40 ng/mL), and insulin (5 µg/mL) with 10% FBS for 28 days and then routinely stained with Oil Red O (Sigma) to identify neutral triglycerides and lipids that are present when cells differentiate into adipocytes. Since MSC were sourced commercially and their multipotency is already well established by their tri-potential differentiation into osteogenic, adipogenic and chondrogenic lineages, in this study we opted to only look at osteogenic and adipogenic differentiation potential as a way to confirm their multipotency. MSC used in this study had been immunophenotyped by the manufacturer and characterized by flow-cytometry and express cell-surface protein profile positive for CD29, CD34, CD44 and Sca-1 (> 70%) but negative for CD117 (< 5%). MSC were activated by stimulating them for 24 h with proinflammatory cytokines IFN-γ (500 U) (R&D Systems, Minneapolis, MN) and TNF-α (50, 500 and 5000 U) (R&D Systems) alone or by a proinflammatory cytokine cocktail of IFN-γ (500 U) and TNF-α (50, 500 and 5000 U).

**Gene expression.** For the gene expression studies 2 x 10^5^ of MSC were seeded into plates and cultured for 24 h prior to treatment with either media alone or proinflammatory cytokines IFN-γ/TNF-α or with a cytokine cocktail for 24 h and RNA was extracted using the RNeasy mini kits (Qiagen, Hilden, Germany). The cDNA was prepared using the High Capacity cDNA Reverse Transcriptase kit (Applied Biosystems, Melbourne, Australia) and generated using the iCycler (Biometra, Victoria, Australia). Gene expression was determined by real time qPCR using SYBR green master mix. qPCR was carried out using the Light Cycler 480 (Roche, NSW, Australia). The relative expression levels of varied genes were calculated using a mathematical model based on the individual qPCR primer efficiencies and the quantified values were normalized against the housekeeping gene RPL13A.

**Microencapsulation.** Microencapsulation procedure described in this study was carried out using the air-driven droplet generator (Torsten Steinau Verfahrenstechnik, Berlin, Germany). Prior to encapsulation procedure, the mouse islets were removed from culture flasks and pooled together in a 50 mL falcon tube in serum-free medium. MSC were routinely harvested using 0.25% trypsin-versene solution in order to obtain a single cell suspension and resuspended in serum-free medium. Mouse islets and MSC were mixed together at a ratio of 1:1, and the cells were washed twice with sterile 0.9 % sodium chloride (NaCl) (Baxter Healthcare Pty Ltd., NSW, Australia) followed by centrifugation at 500 rpm for 5 minutes to form a pellet and the supernatant discarded. The cell suspension was then mixed with 2.2% sodium alginate (Batch no: BP-0908-01; UPMVG Pronova, FMC Biopolymer, Sandvika, Norway) in a ratio of 1:6 (cells:alginate) and injected at a rate of 1 mL/min through the droplet generator with air being introduced at 5 L/min and 80 kPa. The microcapsules were then collected on a big sterile 145 mm petri dish containing 30 mL of barium chloride (BaCl_2_) (Sigma, NSW, Australia) gelling solution which was placed at a distance of 10 cm from tip of the nozzle of the encapsulation device. The microcapsules were then incubated in the BaCl_2_ solution for 2 min and then washed five times with 0.9 % NaCl to remove excess BaCl_2_. After repeated washings, the microcapsules were allowed to settle down and the supernatant discarded. The average size of microcapsules was 748.4 ± 4.4μm. The microencapsulated cells were the cultured for a day in RPMI 1640 media prior to *in vitro* or transplantation studies.

**Viability.** Aliquots of encapsulated islets alone and islets co-encapsulated with unstimulated or stimulated MSC were randomly collected from the tissue culture dishes with a Gilson P 1000 pipette under sterile conditions. They were washed twice by resuspending them in 0.5 mL phosphate buffered saline (PBS) followed by careful removal of the supernatant and discarded. Subsequently, 225 μL of PBS and 25 μL of 6-carboxy fluorescein diacetate (6-CFDA) solution (10 mM) (Sigma) were added to encapsulated cells with gentle tapping of the tube at 10 min intervals. The tubes were then left to incubate in darkness at 37 °C in humidified atmosphere at 5 % CO_2_. After 30 min, encapsulated cells were washed twice with 0.5 mL PBS and the supernatant discarded. The encapsulated cells were again resuspended in 200 μL PBS followed by addition of 10 μL of propidium iodide (PI) (Sigma) stock solution (100 μg/mL). The tubes were incubated at room temperature for 5-10 min and transferred under the cover slip on a glass slide. The encapsulated cells were visualised under a Nikon Eclipse IIi fluorescent microscope (Nikon, NSW, Australia) and the percentage of green (live cells) to red (dead cells) was estimated in 100 microcapsules.

**Insulin secretion and content.** Aliquots of encapsulated islets alone or islets co-encapsulated with unstimulated or stimulated MSC were exposed to either 2.8 mM glucose (basal) or 20 mM glucose (stimulus) for one hour at 37 ˚C with gentle agitation. After 1 h, the supernatant was collected and human insulin measured by time resolved fluorescence resonance energy transfer (FRET) as per manufacturer’s protocol (Cisbio, Codolet, France). The remaining pellet was washed in Hanks Balanced Salt solution (HBSS) solution (Sigma) followed by addition of cold acid ethanol and vortexed vigorously to enhance cell lysis. The cell extract was kept at 4 ˚C overnight and the supernatant collected the following day for measuring insulin content by time resolved FRET (Cisbio).

**Transplantation.** Recipient female immunocompetent C57BL/6 mice (10-12 weeks) and female immunodeficient NOD/SCID mice (8-10 weeks) were made diabetic by intraperitoneal injection of streptozotocin (STZ) (Sigma) at 50 mg/kg (for 5 consecutive days on animals fasted overnight) and 70 mg/kg (for 3 consecutive days) respectively 1-2 weeks prior to transplantation. The animals were considered diabetic if the three consecutive blood glucose levels > 20 mmol/L and enrolled in the study. Anaesthesia was induced using 3% isoflurane (Ethical Agents, Manukau, New Zealand) in air and mice were maintained on 1-2% isoflurane in air. Microencapsulated cells were delivered intraperitoneally through a 14G catheter (Terumo, NSW, Australia) via a ventral midline incision made with a scalpel and the peritoneum and abdominal musculature were closed using a horizontal mattress suture and superimposed purse string suture using 3-0 vicryl (Dynek Pty Ltd., NSW, Australia). The skin was closed with stainless steel staples. A preliminary experiment was carried out to determine the minimal islet mass where C57BL/6 and NOD/SCID mice were transplanted with 500 and 1000 IEQ of encapsulated QS islets. Once the minimal islet mass was determined, subsequent experiments were carried out in an allotransplantation setting in C57BL/6 mice with co-encapsulated or co-transplanted MSC. Accordingly, C57BL/6 mice were randomly assigned to one of the following treatment groups: (i) 500 IEQ encapsulated islets alone, (ii) 500 IEQ encapsulated islets co-encapsulated with unstimulated MSC at 1:1 ratio, (iii) 500 IEQ encapsulated islets co-encapsulated with stimulated MSC at 1:1 ratio, (iv) 500 IEQ encapsulated islets co-transplanted with unstimulated MSC at 1:1 ratio and (v) 500 IEQ encapsulated islets co-transplanted with stimulated MSC at 1:1 ratio. Animals were considered normoglycemic if three consecutive blood sugar levels were < 11.1 mmol/L.

**Glucose tolerance tests.** For the C57BL/6 recipients, an intraperitoneal glucose tolerance test (IPGTT) was carried out at day 49 post-transplantation to confirm their ability to produce insulin in response to a glucose challenge. For this, the mice were fasted overnight followed by an intraperitoneal glucose (Sigma) lavage (2 mg/g of 300 mg/mL glucose solution) and BGLs were measured at 0, 15, 30, 60 and 120 min after glucose administration. IPGTT also were carried out on diabetic and non-diabetic control C57BL/6 mice. For the NOD/SCID recipients, an oral glucose tolerance test (OGTT) was performed on overnight fasted animals followed by an oral glucose gavage (3 mg/g of 300 mg/mL glucose solution) and BGL measurements made at 0, 20, 40, 60 and 120 min after glucose administration. OGTT also was carried out on non-diabetic control NOD/SCID mice.

**Graft retrieval and PFO assessment.** At the end of study period mice were euthanized and microcapsules were retrieved by peritoneal lavage with a minimal volume of 2 ml PBS containing 1% bovine serum albumin (BSA) (Sigma). The microcapsules were removed from the peritoneal lavage fluid by gravity separation on bench for 15 min. The residual peritoneal fluid was spun at 3000 rpm to remove immune cells and debris, and then frozen at -80 ^o^C for analysis of cytokines/chemokines. The peritoneal cavity was then flushed with 0.9% NaCl (Baxter) and examined for any remaining microcapsules. Microcapsules were observed under the microscope and degree of PFO was assessed in a blinded fashion using a scoring system. Briefly, the microcapsules were scored as follows: 0 = no overgrowth, 1 = < 25% of microcapsule overgrown, 2 = 25-50% of microcapsule overgrown, 3 = 50-75% of microcapsule overgrown and 4 = > 75% of microcapsule overgrown. The fibrotic score index was calculated for each mouse using the formula (0 x % score 0) + (2 x % score 1) + (4 x % score 2) + (8 x % score 3) + (16 x % score 4), giving a minimum possible fibrotic score of 0 and a maximum possible fibrotic score of 16.
