# Supplementary material for: Co-encapsulation and co-transplantation of mesenchymal stem cells reduces pericapsular fibrosis and improves encapsulated islet survival and function when allografted
Source: Sci Rep. 2017 Aug 30;7:10059. doi: 10.1038/s41598-017-10359-1 (PMC5577272; doi:10.1038/s41598-017-10359-1)
Supplement: Supplementary file 2 — Dataset 1 [file 41598_2017_10359_MOESM2_ESM.doc]

**Co-encapsulation and co-transplantation of mesenchymal stem cells reduces pericapsular fibrosis and improves encapsulated islet survival and function when allografted**

Vijayaganapathy Vaithilingam, PhD^1,*^, Margaret DM Evans, PhD^1^, Denise M Lewy, BSc^1^, Penelope A Bean, BSc^1^, Sumeet Bal, MSc^1^ & Bernard E Tuch, MD, PhD^1,2^

^1^Biomedical Manufacturing Research Program, Commonwealth Scientific and Industrial Research Organization (CSIRO), Manufacturing Flagship, North Ryde, New South Wales, Australia.

^2^Australian Foundation for Diabetes Research, Sydney, New South Wales, Australia, previously at CSIRO Manufacturing Flagship, North Ryde, New South Wales, Australia.

**
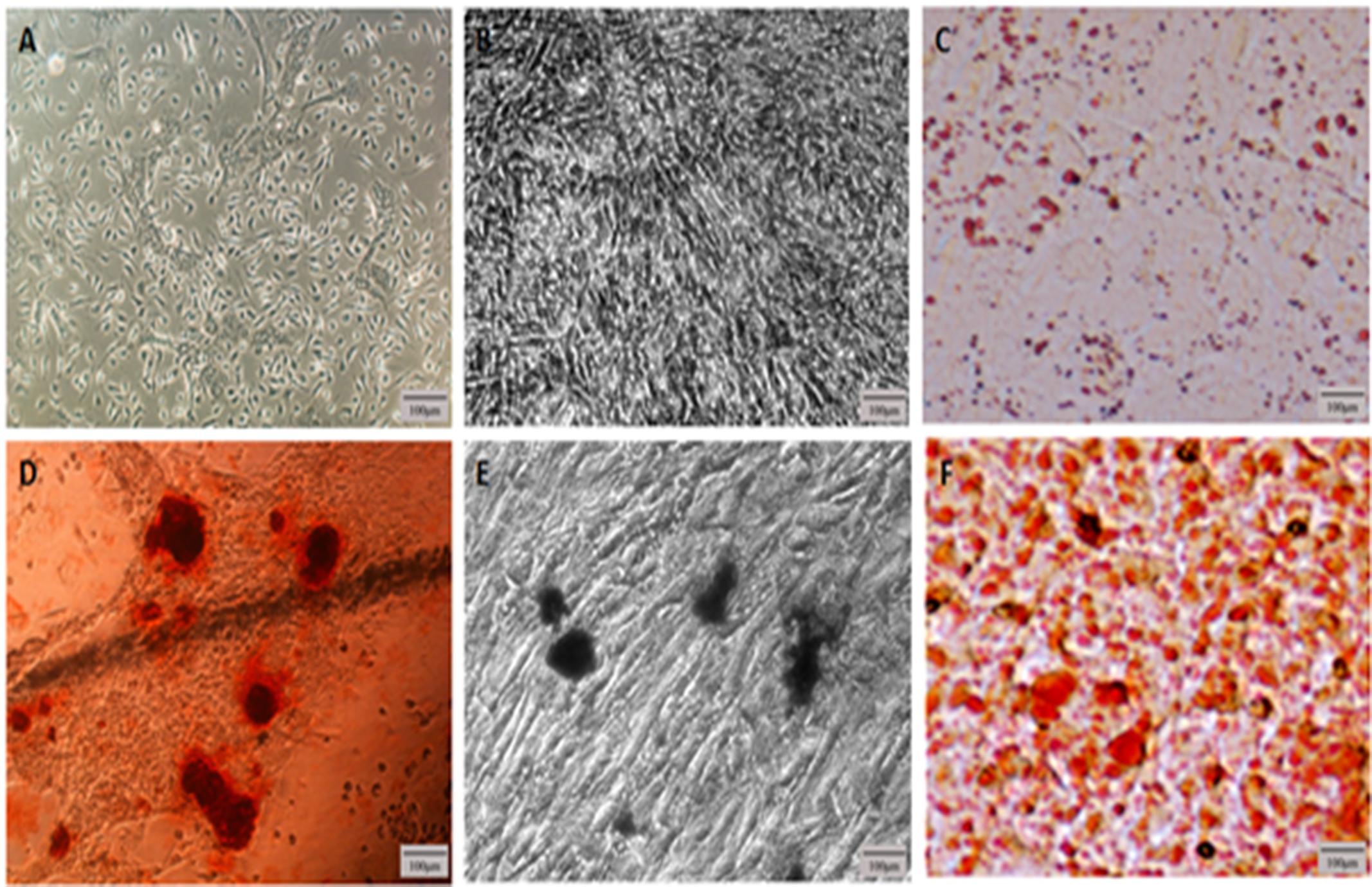
**

**Supplementary Figure 1. Stemness of MSC.** The multipotency of MSC was confirmed by differentiation into osteogenic and adipogenic lineage using an osteogenic and adipogenic culture regime. Representative images of MSC differentiated for 28 days in osteogenic culture condition and stained with Alizarin red (D) and Von Kossa (E); adipocyte culture regime and stained with Oil Red O (F); controls were MSC cultured in usual culture regime and stained for Alizarin red (A), Von Kossa (B) and Oil Red O (C) respectively. Scale bar = 100 μm

**
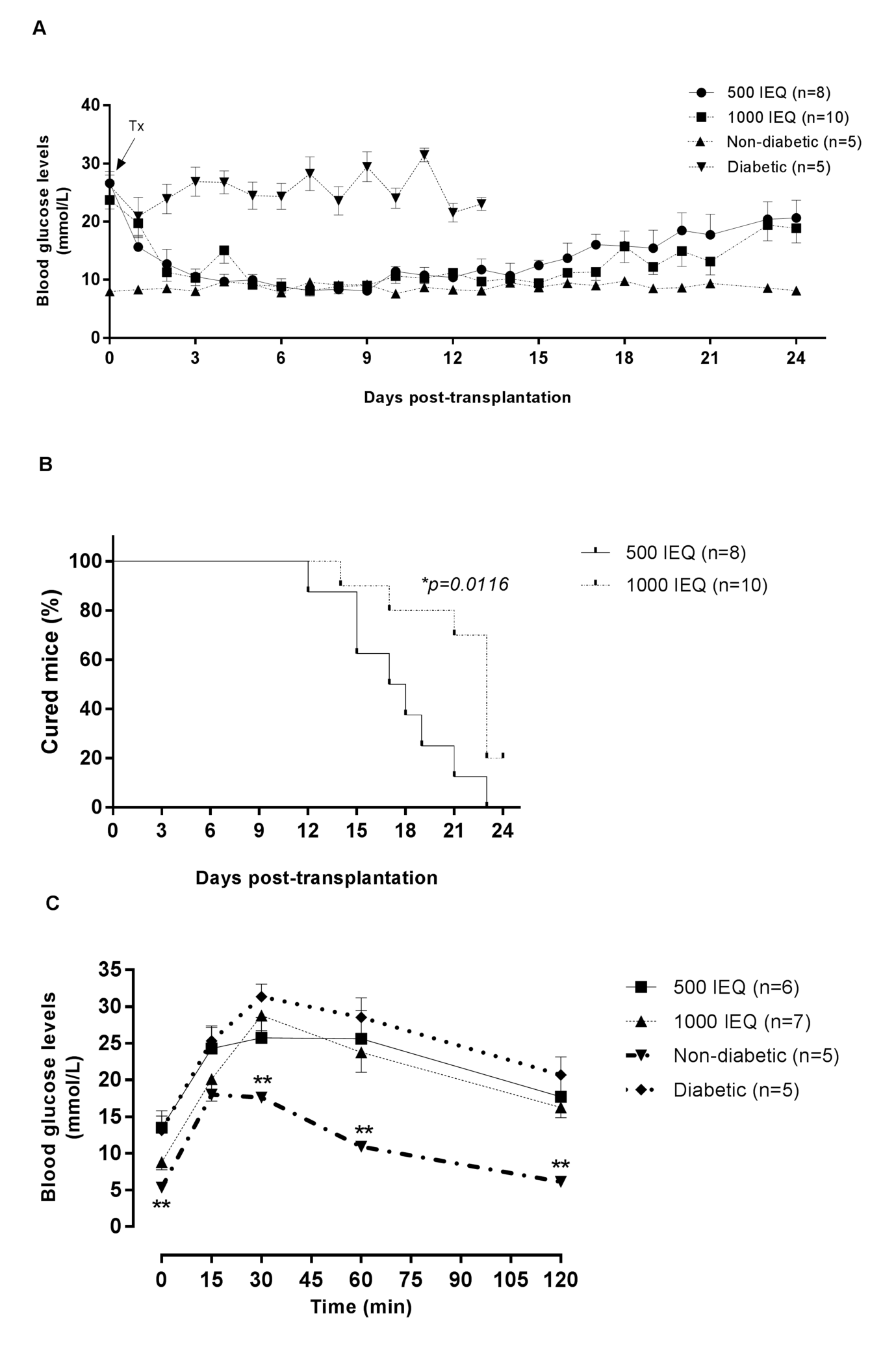
**

**Supplementary Figure 2. Determination of minimal islet mass.** Random average blood glucose levels (BGLs) of diabetic C57BL/6 mice allotransplanted intraperitoneally with 500 or 1000 IEQ of encapsulated QS mouse islets (A); Values = mean ± SEM. Percentage of mice that became normoglycemic when transplanted with 500 or 1000 IEQ encapsulated islets (B); *p < 0.05 (Kaplan-Meier survival analysis [log-rank]). IPGTT carried out at day 24 post-transplantation (C); Values = mean ± SEM; **p < 0.01 at time 0 where the blood glucose levels for non-diabetic controls < 500 IEQ, and diabetic controls and **p < 0.01 at time 30, 60 and 120 where the blood glucose levels for non-diabetic controls < 500 IEQ, 1000 IEQ and diabetic controls (ANOVA with posthoc Duncan’s Multiple Comparison Test).

**
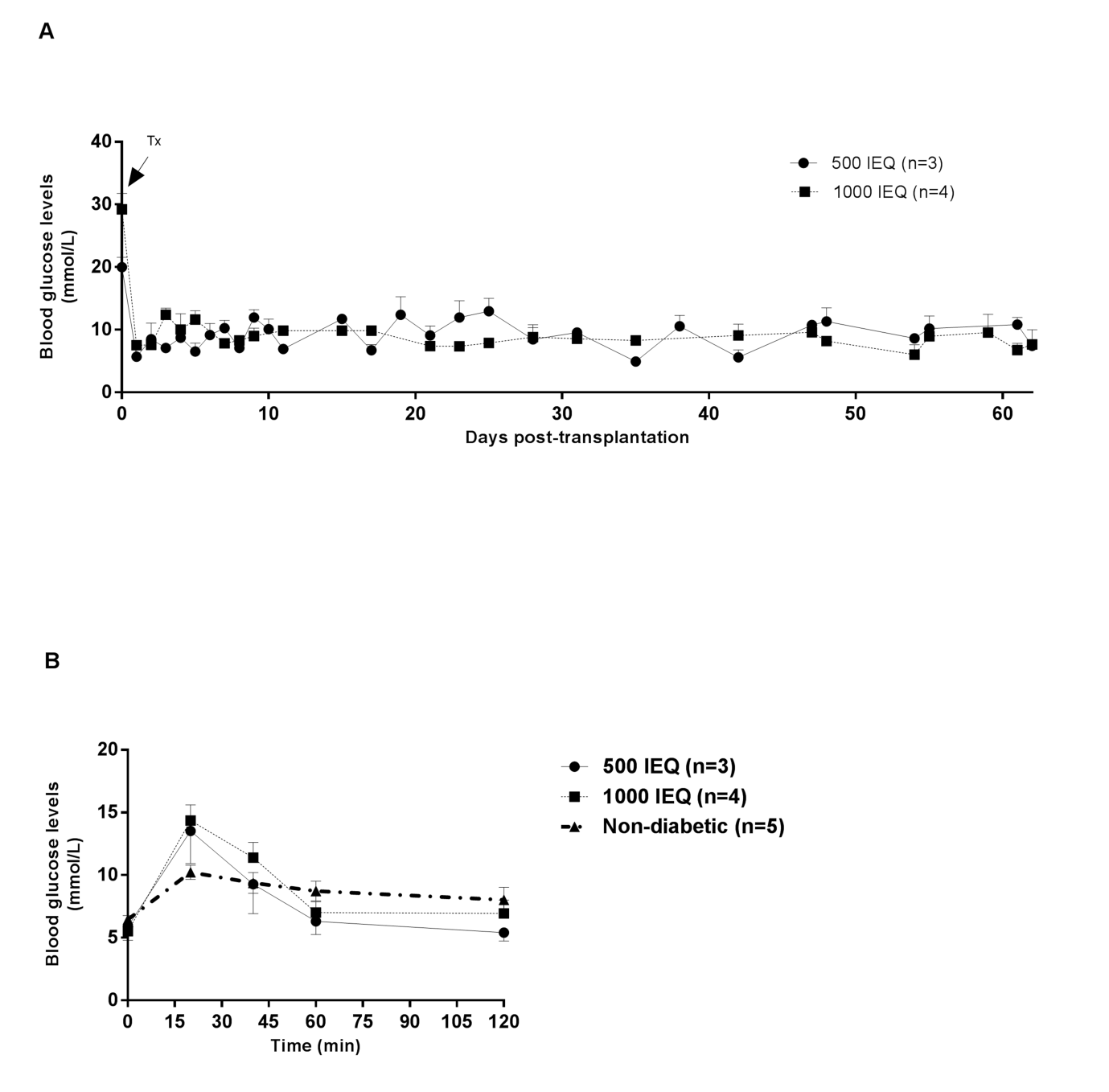
**

**Supplementary Figure 3. Transplantation of encapsulated islets into diabetic NOD/SCID mice.** Random average blood glucose levels of diabetic NOD/SCID mice transplanted intraperitoneally with 500 or 1000 IEQ of encapsulated QS mouse islets (A); Values = mean ± SEM. Oral glucose tolerance test (OGTT) carried out at day 60 post-transplantation (B); Values = mean ± SEM; p > 0.05 at all time points when compared between 500 or 1000 IEQ with non-diabetic controls (ANOVA with posthoc Duncan’s Multiple Comparison Test).


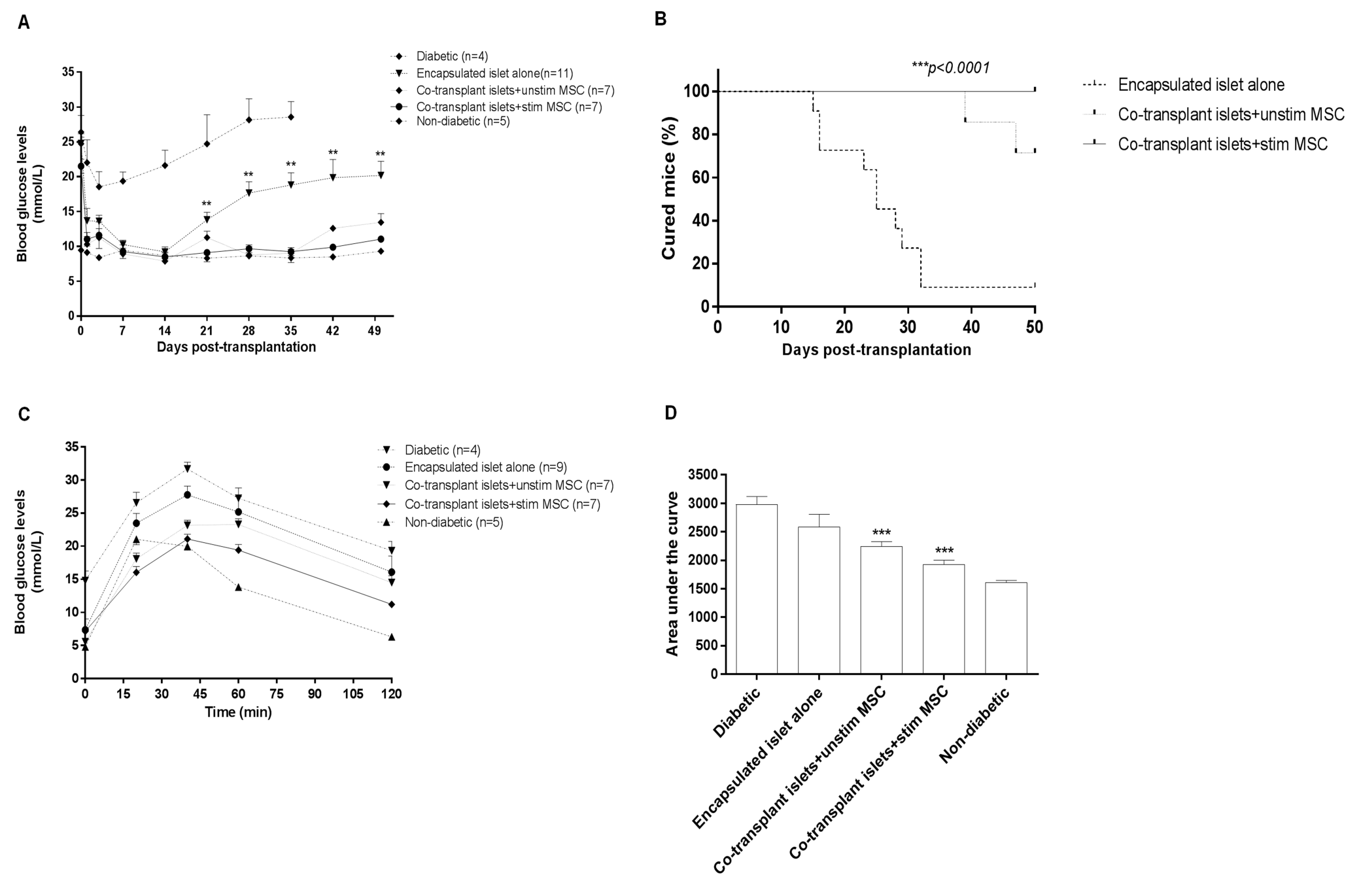


**Supplementary Figure 4. Transplantation of islets co-transplanted with stimulated or unstimulated MSC into diabetic C57BL/6 mice.** Random average blood glucose levels of diabetic C57BL/6 mice allotransplanted intraperitoneally with 500 IEQ of islets co-transplanted with stimulated or unstimulated MSC (A); Values = mean ± SEM; **p < 0.01 for BGLs at days 21, 28, 35, 42 and 50 where encapsulated islets alone > co-transplanted islet with unstimulated or stimulated MSC (ANOVA with posthoc Duncan’s Multiple Comparison Test). Percentage of mice that became normoglycemic after being transplanted with 500 IEQ of encapsulated islets co-transplanted with stimulated or unstimulated MSC; ***p < 0.0001 (Kaplan-Meier survival analysis [log-rank]). IPGTT carried out at day 49 post-transplantation (C); Values = mean ± SEM. Area under the curve (AUC) for IPGTT values (D); Values = mean ± SEM; ***p < 0.0001 for AUC where co-transplanted islets + stimulated MSC < diabetic controls and encapsulated islets alone; and ***p < 0.0001 for AUC where co-transplanted islets + unstimulated MSC < diabetic controls and encapsulated islets alone; and co-transplanted islets + unstimulated MSC > non-diabetic controls. There is no significant difference for AUC between co-transplanted islets + stimulated MSC and non-diabetic controls (ANOVA with posthoc Duncan’s Multiple Comparison Test).


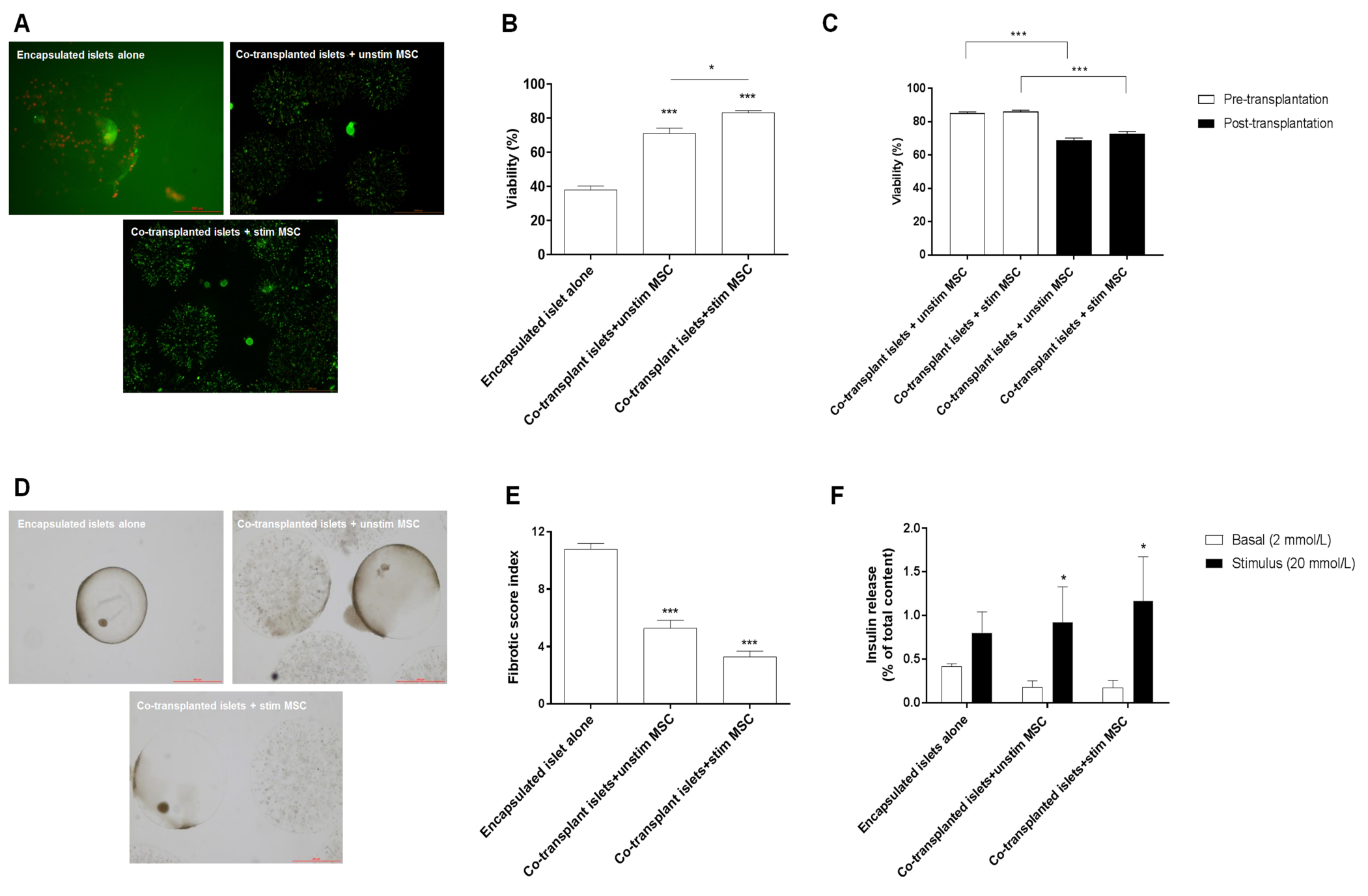


**Supplementary Figure 5. Assessment of grafts retrieved from C57BL/6 transplanted with islets co-transplanted with stimulated or unstimulated MSC.** Representative viability images of grafts retrieved from different treatment groups at day 50 post-transplantation (A) (Scale bar = 1000 μm). Percentage viability of encapsulated islets retrieved from different treatment groups at day 50 post-transplantation (B). Values = mean ± SEM (n=100 islets for each treatment group); ***p < 0.0001 for viability where co-transplanted islets + unstimulated MSC and co-transplanted islets + stimulated MSC > encapsulated islets alone (ANOVA with posthoc Duncan’s Multiple Comparison Test) and *p < 0.05 for viability when compared between co-transplanted islets + unstimulated MSC and co-transplanted islets + stimulated MSC (Student’s t-test). Percentage viability of encapsulated MSC retrieved from different treatment groups at day 50 post-transplantation (C). Viability of co-transplanted MSC pre-transplantation (unstimulated vs stimulated: 85.1 ± 0.8 vs 86.1 ± 0.8%) and at day 50 post-transplantation (unstimulated vs stimulated: 68.9 ± 1.4 vs 72.6 ± 1.3%). Values = mean ± SEM (n=100 encapsulated MSC for each treatment group); ***p < 0.001 for viabilities of both unstimulated and stimulated MSC where viability of pre-transplanted group > post-transplanted group (ANOVA with posthoc Duncan’s Multiple Comparison Test). Representative images of grafts retrieved from different treatment groups showing the degree of PFO at 50 days post-transplantation (D) (Scale bar = 500 μm). Extent of PFO on retrieved grafts represented as fibrotic score index, on a scale of 0 (no fibrotic overgrowth) to 16 (complete fibrotic overgrowth) (E); Values = mean ± SEM (n=6-9); ***p < 0.0001 for fibrotic score index where encapsulated islets alone > co-transplanted islets with unstimulated or stimulated MSC (ANOVA with posthoc Duncan’s Multiple Comparison Test). *Ex-vivo* static stimulation studies on grafts retrieved from different treatment groups (F); Values = mean ± SEM (n=5-7); *p < 0.05 when compared between basal (2 mmol/L) and stimulus (20 mmol/L) for grafts retrieved from co-transplanted groups containing either unstimulated or stimulated MSC (Student’s t-test).


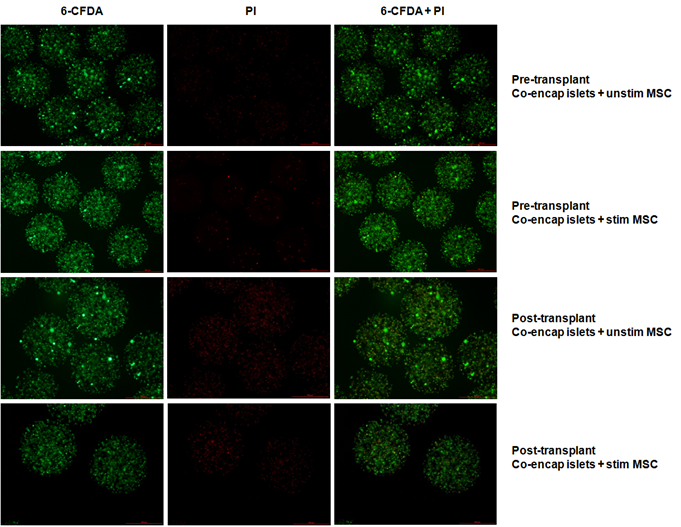


**Supplementary Figure 6.** **Viability of MSC co-encapsulated with islets**. Representative viability images of MSC in different treatments groups measured pre-transplantation and at day 50 post-transplantation (Green; 6-CFDA – live cells & Red; PI – dead cells). Scale bar = 500 μm.

**
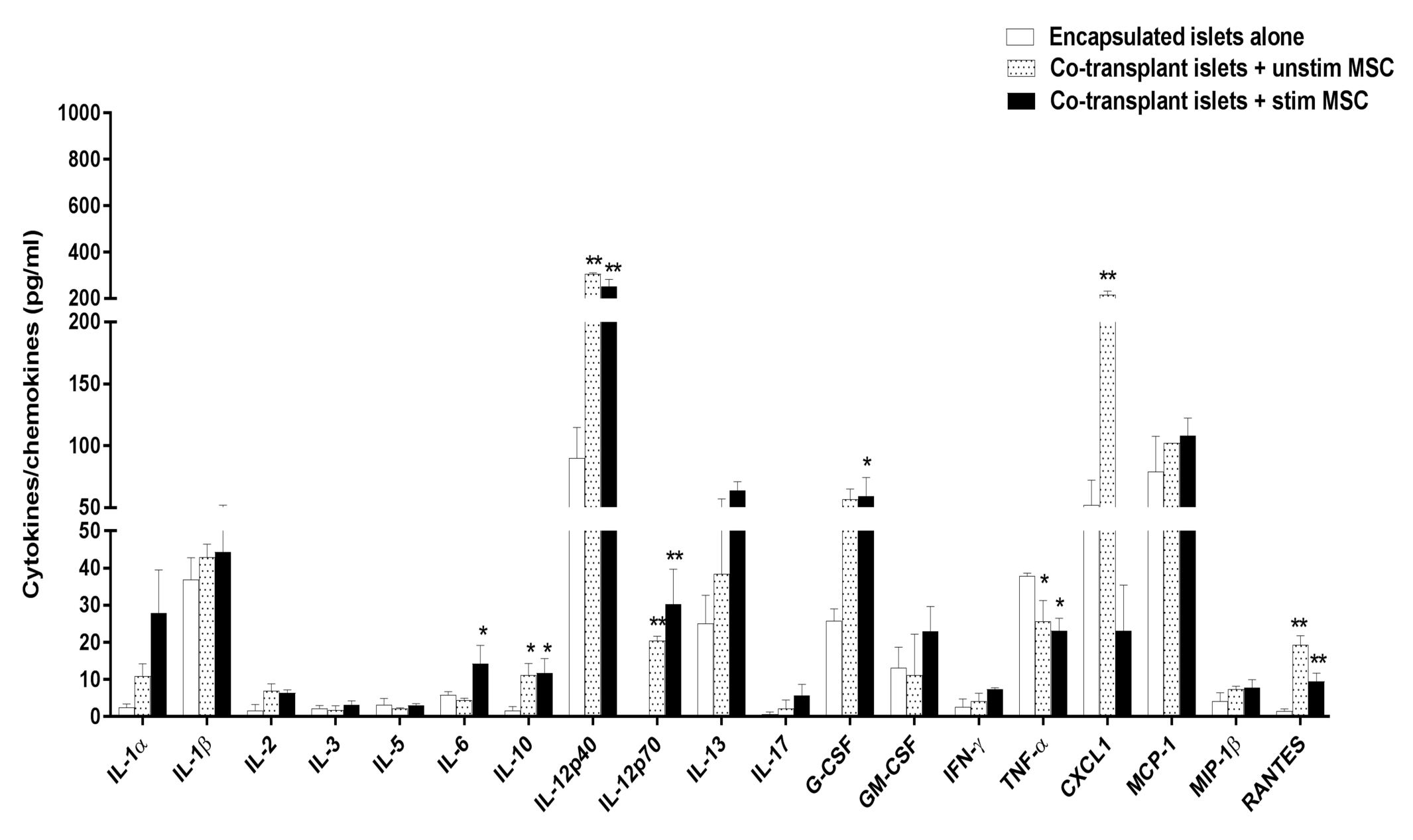
**

**Supplementary Figure 7. Analysis of peritoneal cytokines/chemokines.** Levels of cytokines/chemokines measured in the peritoneal fluid of C57BL/6 mice co-transplanted with encapsulated islets and encapsulated stimulated or unstimulated MSC. Values = mean ± SEM (n=4-5); *p < 0.05, **p < 0.01 and for cytokines/chemokines levels where islets co-transplanted with unstimulated or stimulated MSC > encapsulated islets alone (ANOVA with posthoc Duncan’s Multiple Comparison Test).
